# Overlapping and Non-overlapping Functions of Condensins I and II in Neural Stem Cell Divisions

**DOI:** 10.1371/journal.pgen.1004847

**Published:** 2014-12-04

**Authors:** Kenji Nishide, Tatsuya Hirano

**Affiliations:** Chromosome Dynamics Laboratory, RIKEN, Hirosawa, Wako, Saitama, Japan; Geisel School of Medicine at Dartmouth, United States of America

## Abstract

During development of the cerebral cortex, neural stem cells (NSCs) divide symmetrically to proliferate and asymmetrically to generate neurons. Although faithful segregation of mitotic chromosomes is critical for NSC divisions, its fundamental mechanism remains unclear. A class of evolutionarily conserved protein complexes, known as condensins, is thought to be central to chromosome assembly and segregation among eukaryotes. Here we report the first comprehensive genetic study of mammalian condensins, demonstrating that two different types of condensin complexes (condensins I and II) are both essential for NSC divisions and survival in mice. Simultaneous depletion of both condensins leads to severe defects in chromosome assembly and segregation, which in turn cause DNA damage and trigger p53-induced apoptosis. Individual depletions of condensins I and II lead to slower loss of NSCs compared to simultaneous depletion, but they display distinct mitotic defects: chromosome missegregation was observed more prominently in NSCs depleted of condensin II, whereas mitotic delays were detectable only in condensin I-depleted NSCs. Remarkably, NSCs depleted of condensin II display hyperclustering of pericentric heterochromatin and nucleoli, indicating that condensin II, but not condensin I, plays a critical role in establishing interphase nuclear architecture. Intriguingly, these defects are taken over to postmitotic neurons. Our results demonstrate that condensins I and II have overlapping and non-overlapping functions in NSCs, and also provide evolutionary insight into intricate balancing acts of the two condensin complexes.

## Author Summary

The cerebral cortex is built up of numerous neurons and cells supporting them, most of which originate from neural stem cells (NSCs). NSCs divide symmetrically to produce themselves and asymmetrically to generate neurons, and both types of divisions reply on faithful segregation of chromosomes into daughter cells. In the current study, we study the functions of evolutionarily conserved chromosome regulators, known as condensin I and condensin II, during development of the cerebral cortex in mice. We find that condensins I and II have both overlapping and non-overlapping functions in NSC divisions and survival: loss of either one of condensins causes distinct abnormalities in the process of chromosome segregation. Remarkably, loss of condensin II, but not of condensin I, also alters chromosome architecture during non-dividing stages. Our results demonstrate convincingly that an intricate balance between condensins I and II plays a crucial role in NSC divisions. It will be of great interest to test in the future whether such balancing acts of the two condensin complexes might be misregulated in tumorigenic NSCs that undergo uncontrolled cell divisions.

## Introduction

**Figure 1:**
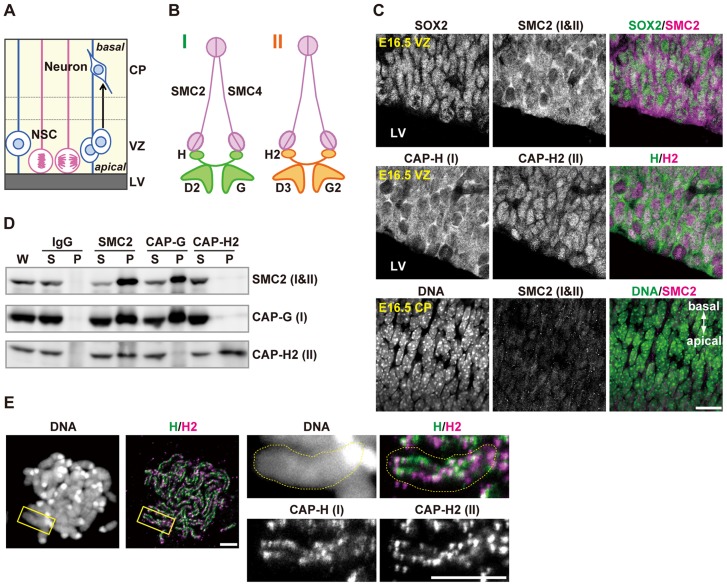
Condensins I and II are both present in NSCs. (**A**) Schematic representation of NSC division and neural differentiation. NSCs possess radial fibers along apico-basal polarity and exhibit cell cycle-dependent nuclear movement (i.e., interkinetic nuclear migration). LV, VZ and CP indicate the lateral ventricle, the ventricular zone and the cortical plate, respectively. (**B**) Subunit composition of the condensin complexes. Both complexes share the same pair of SMC core subunits (SMC2 and SMC4). Condensin I possesses three non-SMC subunits (CAP-D2, CAP-G and CAP-H) whereas condensin II has a distinct set of non-SMC subunits (CAP-D3, CAP-G2 and CAP-H2). (**C**) Frozen sections of embryonic brains were immunolabeled with antibodies against SOX2 (an NSC marker) and condensin subunits. Areas containing the VZ were labeled with antibodies against SOX2 and SMC2 (upper panels) or against CAP-H and CAP-H2 (middle panels). An area containing the CP was labeled with an SMC2 antibody. DNA was counterstained with Hoechst (lower panes). The data shown are from a single representative experiment out of three repeats. Sections from three different embryos were used. Bar, 20 µm. (**D**) Brain extracts prepared from C57BL/6J embryos at E12.5 are subjected to immunoprecipitation assay with antibodies against condensin subunits and control IgG as indicated on the top. The precipitates (P) along with whole cell extracts (W; 5% of input) and non-precipitated supernatants (S; 5% of input) were analyzed by western blotting. (**E**) Metaphase spreads were prepared from NSC cultures and immunolabeled with specific antibodies against CAP-H and CAP-H2. DNA was counterstained with Hoechst. Maximum intensity projections are shown. The right four panels indicate close-up views of a representative chromosome (indicated by the yellow rectangles in the left panels). The perimeter of the chromosome is indicated by the dotted lines. The data shown are from a single representative experiment out of two repeats. Bars, 5 µm.

The formation of the cerebral cortex requires a stringent control of cell proliferation and differentiation [1], [2]. At the early stages of cortical development, NSCs in the ventricular zone (VZ) adjacent to the lateral ventricle (LV) divide symmetrically to generate two daughter NSCs for expanding the cell population (Fig. 1A). NSCs then undergo asymmetric divisions to give rise to pairs of a neuron and an NSC. NSCs also generate lineage-committed intermediate progenitor cells that contribute to further amplification of the number of neurons. Newly born neurons migrate away from the VZ into the cortical plate (CP) in a birthdate-dependent manner. Thus, both proliferation and differentiation of NSCs rely on a series of symmetrical and asymmetrical cell divisions, where two daughter cells must receive a complete set of chromosomes. Despite its importance, however, the basic mechanism of chromosome inheritance is often overlooked in the studies of NSCs with a few exceptions [3].

Faithful assembly and segregation of chromosomes during mitosis rely on a class of multisubunit protein complexes, known as condensins [4]. Many eukaryotic organisms have two different condensin complexes (condensin I and condensin II) although a limited number of organisms including fungi have condensin I only. It remains unknown why the two condensin complexes are widespread among eukaryotes. Surprisingly, it has been shown that condensin II is not essential for mitosis in several model organisms [5], [6], and that condensin II has specialized non-mitotic functions, such as polytene chromosome disassembly in *Drosophila melanogaster* [7] and stress response in *Arabidopsis thaliana* [8]. In *Xenopus* egg extracts, condensin II has only a minor contribution to mitotic chromosome architecture [9]. Moreover, MCPH1, whose dysfunctions cause primary microcephaly in humans [10], has been shown to function as a potent inhibitor of condensin II [11], implicating that the action of condensin II may strongly be suppressed during cortical development. All these data suggest that condensin I is the major player in mitotic chromosome assembly and segregation among eukaryotes. A notable exception to this view exists, however: condensin II plays a dominant role over condensin I during early embryonic divisions in *Caenorhabditis elegans* [12]. This unique feature could be due to the occurrence of the holocentric chromosome structure in this particular organism. In vertebrates, experiments involving functional knockdown of condensin subunits have been limited to those using tissue culture cells [13]–[15], and no systematic genetic studies at an organismic level have been reported so far. Equally important, it has been difficult to follow the physiological fate of condensin-depleted cells in studies using tissue culture cells.

In the current study, we have generated several strains of conditional knockout (cKO) mice to investigate the roles of condensins I and II in NSC divisions during cortical development. Contrary to the prediction described above, we find that condensins I and II are both essential to ensure NSC divisions and survival: loss of either one of condensins induces DNA damage and p53 nuclear accumulation, eventually leading to apoptosis. Simultaneous depletion of both condensins causes a severer set of defective phenotypes, indicating that the two complexes have overlapping functions. Remarkably, however, depletion of individual condensin causes distinct phenotypes, indicating that they have non-overlapping functions, too. Most notably, it is found that pericentromeric heterochromatin regions form abnormally large clusters in condensin II-depleted NSCs and postmitotic neurons. Our results clearly delineate the essential and distinct functions of the two condensin complexes in NSC divisions during cortical development.

## Results

### Condensins I and II are both present in NSCs during cortical development

We first examined the expression pattern of condensins I and II in the developing brain by immunohistochemical analyses using specific antibodies against mouse condensin subunits (Fig. 1A and 1B). SOX2 was used as a marker for NSCs. We found that SMC2, a subunit common to condensins I and II, was detected in SOX2-positive cells in the VZ (Fig. 1C, upper panels). Similarly, condensin I-specific subunit (CAP-H) and condensin II-specific subunit (CAP-H2) were both present in the VZ (Fig. 1C, middle panels). CAP-H was mainly detectable in the cytoplasm whereas CAP-H2 localized to the nucleus in NSCs during interphase, in agreement with previous reports using tissue culture cells [16] and mouse oocytes [17]. Unlike in the VZ, however, SMC2 was rarely detectable in the CP at embryonic day 16.5 (E16.5) (Fig. 1C, lower panels). Immunoprecipitation analyses using extracts of developing brains showed that an antibody against SMC2 co-precipitated both condensin I-specific subunit (CAP-G) and condensin II-specific subunit (CAP-H2)(Fig. 1D). Conversely, SMC2 was efficiently co-precipitated with an anti-CAP-G antibody, but barely with an anti-CAP-H2 antibody. Moreover, the subcellular localization of SMC2 resembled that of CAP-H (Fig. 1C), implicating that the amount of condensin I exceeds that of condensin II in NSCs. Finally, we found that CAP-H and CAP-H2 localized to distinct but partially overlapping regions on mitotic chromosomes in NSCs (Fig. 1E). These results suggest that condensins I and II are present in NSCs but are differentially regulated during cortical development, and that they are largely absent in neurons.

### Condensins I and II are both required for proper formation of the cerebral cortex

**Figure 2:**
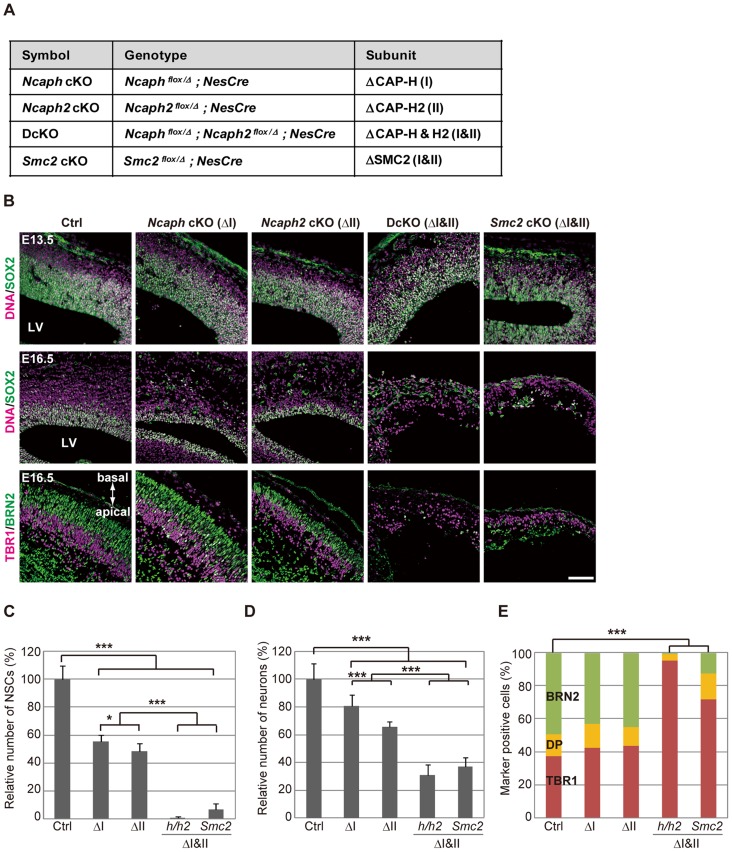
Condensins I and II are both required for proper development of the cerebral cortex. (**A**) Genotypes of mice used in the current study. The heterozygotes of *Ncaph* or *Ncaph2* mutants in the *NesCre* background were used as a control (Ctrl). (**B**) Frozen sections of embryonic brains from cKO mice at E13.5 or E16.5 were immunolabeled with cell-type specific markers: SOX2 for NSCs; TBR1 for deep-layer neurons; BRN2 for upper-layer neurons. DNA was counterstained with Hoechst in the upper and middle panels. The data shown are from a single representative experiment out of three repeats. Sections from at least two different embryos of each genotype were analyzed. Bar, 100 µm. (**C**) The numbers of SOX2-positive cells were measured in the dorsal area of cortices in control and cKO mice. Data were obtained from four independent sections from two different embryos and normalized to the mean number of NSCs in control mice as 100%. Bars indicate the mean and SD. * *P*<0.05, *** *P*<0.001 (*t*-test with a Holm correction for multiple comparisons). (**D**) The numbers of cells present in the CP were measured. Data were obtained and analyzed as in (**C**). (**E**) The ratios of TBR1-positive, BRN2-positive, and double-positive (DP) cells were measured in the CP at E16.5. Data were obtained from two different embryos (at least 600 neurons scored). *** *P*<0.001 (Chi-squared test).

To elucidate the functions of condensins I and II in NSCs, we generated four strains of cKO mice in which genes encoding condensin subunits could be deleted by a Cre recombinase (Fig. 2A; for details, see S1, S2, S3 Figures). We were able to obtain none of homozygotes with complete deletion alleles for *Smc2*, *Ncaph* (encoding the condensin I-specific subunit CAP-H) or *Ncaph2* (encoding the condensin II-specific subunit CAP-H2) at E12.5 (S4A and S4B Figure), indicating that condensins I and II are both essential for early embryonic development. We then used *NesCre* transgenic mice that express Cre recombinase under the control of the NSC-specific Nestin promoter [18], and attempted to conditionally deplete condensin subunits in developing brains. It was confirmed that the targeted subunits were successfully depleted in the VZ in *Ncaph* cKO (*Ncaph ^flox/Δ^*; *NesCre*), *Ncaph2* cKO (*Ncaph2^flox/Δ^*; *NesCre*), double cKO (DcKO, *Ncaph ^flox/Δ^*; *Ncaph2^flox/Δ^*; *NesCre*) and *Smc2* cKO mice (*Smc2^flox/Δ^*; *NesCre*), as judged by immunohistochemical analyses (S1D, S2D and S3D Figures). Although some of the targeted subunits were still faintly detectable in the VZ at E13.5, probably because of their long half-lives, they were almost completely depleted by E16.5.

Next we examined the brain structures in the four strains of cKO mice at various embryonic stages. At E13.5, the cerebral cortices were apparently normal, and SOX2-positive NSCs were retained in the VZ in all mutant mice (Fig. 2B, upper panels; S4C Figure, upper panels). The structures were, however, severely disorganized by E16.5 in DcKO and *Smc2* cKO mice: their cortices were extremely thin and SOX2-positive NSCs largely disappeared (Fig. 2B, middle panels, and Fig. 2C; S4CF, middle panels). Neurons in the CP were clearly reduced in DcKO and *Smc2* cKO mice compared to control mice (Fig. 2D). Immunohistochemical analyses using cell-type specific markers [19] revealed that the BRN2-positive upper-layer (late born) neurons were depleted more severely than the TBR1-positive deep-layer (early born) neurons (Fig. 2B, lower panels; Fig. 2E). Compared with DcKO and *Smc2* cKO mice, the morphological defects observed in *Ncaph* cKO and *Ncaph2* cKO mice were relatively mild at E16.5 (Fig. 2B, middle panels; S4C Figure, middle panels). Nevertheless, the numbers of both NSCs and neurons were substantially decreased (Fig. 2C and 2D), suggesting that condensins I and II have non-redundant functions in NSC divisions. By E19.5, *Ncaph* cKO and *Ncaph2* cKO mice displayed highly disorganized cerebral cortices, whereas *Smc2* cKO mouse no longer possessed recognizable cortex (S4C Figure, lower panels). On the basis of these results, we conclude that the functions of both condensins I and II in NSCs are essential for cortical development.

### Defective chromosome segregation leads to DNA damage-induced apoptosis

**Figure 3:**
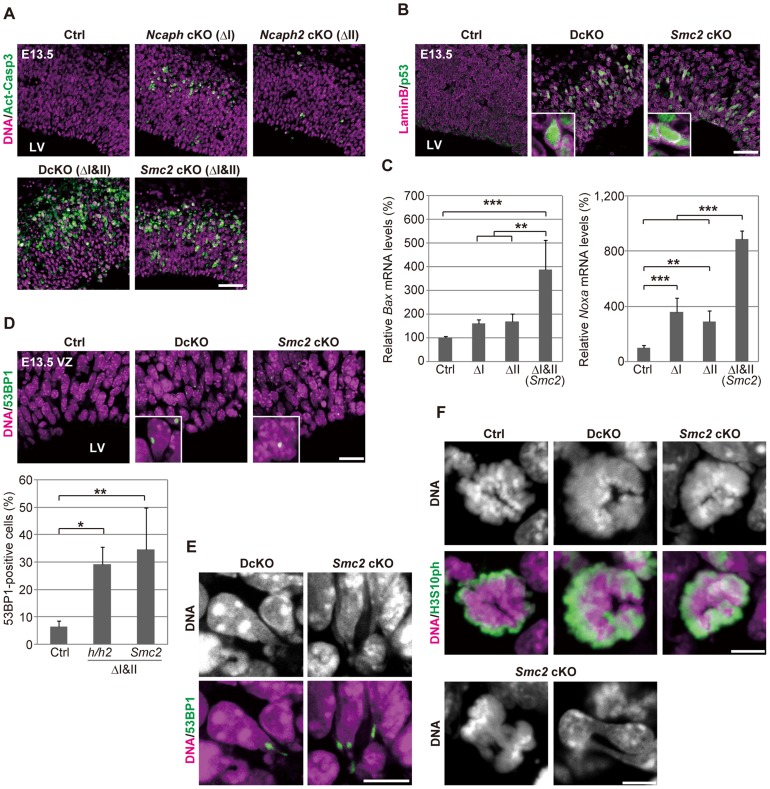
Defective chromosome segregation leads to DNA damage-induced apoptosis in NSCs depleted of both condensins. (**A**) Frozen sections of embryonic brains at E13.5 were immunolabeled with an antibody against an active form of caspase 3 (Act-Casp3), an indicator of apoptotic cell death, and counterstained with Hoechst. The data shown are from a single representative experiment out of three repeats. Sections from at least two different embryos of each genotype were analyzed. Bar, 50 µm. (**B**) Frozen sections were immunolabeled with antibodies against lamin B and p53. The insets show close-ups of p53-positive nuclei. The data shown are from a single representative experiment out of three repeats. Sections from at least two different embryos of each genotype were analyzed. Bar, 50 µm. (**C**) Real-time RT-PCR analysis was performed to determine mRNA levels of two p53 target genes (*Bax* and *Noxa*) in the cortices of cKO mice at E13.5. The mRNA levels were normalized to the internal control (*Gapdh* mRNA level). Data were obtained from four experiments and show the mean mRNA levels of Ctrl mice as 100%. Bars indicate the mean and SD. ** *P*<0.01, *** *P*<0.001 (*t*-test with a Holm correction for multiple comparisons). (**D**) Frozen sections were immunolabeled with a 53BP1 antibody to visualized damaged DNA foci in interphase nuclei. The insets show close-ups of 53BP1-positive nuclei. The panels shown are from a single representative experiment out of three repeats. Sections from at least two different embryos of each genotype were analyzed. Bar, 20 µm. The percentages of 53BP1-positive nuclei in the VZ were measured in the dorsal area of cortices, and plotted in the bottom. Data were obtained from four independent sections from two different embryos. The bars indicate the mean and SD. * *P*<0.05, ** *P*<0.01 (*t*-test with a Holm correction for multiple comparisons). (**E**) Shown are close-up images of cells with tail-like structures from the same experiments as (**D**). Bar, 10 µm. (**F**) Mitotic cells were identified as H3S10ph-positive cells on the apical surface of the VZ (middle panels), and their chromosome morphology was visualized with Hoechst stain (upper panels). Also shown are chromatin bridges in anaphase or postmitotic cells (lower panels) in the same region. The data shown are from a single representative experiment out of three repeats. Sections from at least two different embryos of each genotype were analyzed. Maximum intensity projections are shown. Bars, 5 µm.

The rapid decrease in the number of NSCs in DcKO and *Smc2* cKO mice prompted us to test for possible occurrence of apoptotic cell death. We detected massive activation of caspase 3 (Fig. 3A) and chromatin fragmentation as judged by TUNEL assay (S4D Figure) in developing cortices at E13.5, demonstrating that apoptosis indeed occurred upon simultaneous depletion of condensins I and II. Fewer apoptotic cells were detected in *Ncaph* cKO and *Ncaph2* cKO mice (Fig. 3A), consistent with their milder phenotypes in terms of NSC loss. Notably, cells with active caspase 3 were detected toward the basal side of the VZ, implicating that apoptosis was executed during interkinetic nuclear migration [20]. We also found that nuclear accumulation of p53 was readily detectable in DcKO and *Smc2* cKO mice at E13.5 (Fig. 3B), and that p53 target genes involved in apoptosis (*Bax* and *Noxa*) [21], [22] were up-regulated in the brain of cKO mice (Fig. 3C). Foci of 53BP1, a protein involved in non-homologous end joining [23], [24], were also detectable in cKO mice depleted of both condensins I and II (Fig. 3D). Because the expression of p21 was reported to be suppressed in NSCs [25]–[27], these results suggest that DNA damage elicits p53 nuclear accumulation and thereby induces apoptosis, rather than G1/S arrest, in these mice. Moreover, we noticed that postmitotic cells with tail-like structures, indicative of defective chromosome segregation, were frequently positive for 53BP1 (Fig. 3E; 32.5% in DcKO mice and 33.9% in *Smc2* cKO mice; >40 cells were scored per experiment), raising the possibility that defects in chromosome segregation provides a potential source of DNA damage.

We found that mitotic chromosomes in DcKO and *Smc2* cKO mice were highly disorganized at prometaphase (Fig. 3F; 0% in control mice, 87.8% in DcKO mice and 88.6% in *Smc2* cKO mice; >40 cells were scored per experiment): fuzzy masses of chromatin were observed, in which individual chromosomes were no longer discernible from each other. Although it had been reported that RNAi-mediated depletion of *Smc2* induces metaphase arrest in mouse embryonic stem cells [28], such arrest was not observed in NSCs in DcKO and *Smc2* cKO mice. The cells with abnormal chromosomes progressed through mitosis, exhibiting chromosome bridges in anaphase and dumbbell-shaped nuclei in subsequent G1 phase (Fig. 3F; 0% in control mice, 76.0% in DcKO mice and 80.0% in *Smc2* cKO mice; 50 cells were scored per experiment). To further analyze the missegregation phenotypes, NSC cultures were prepared from *Smc2* cKO mouse brains. The cells displayed limited proliferation capacities as judged by neurosphere formation (S5A and S5B Figure) and cell population sizes (S5C Figure). Remarkably, the epitope of γH2A.X was often detectable along the tail-like structure connecting two daughter nuclei (S5D Figure).

### Condensins I and II differentially contribute to mitotic progression, chromosome morphology and cell survival

**Figure 4:**
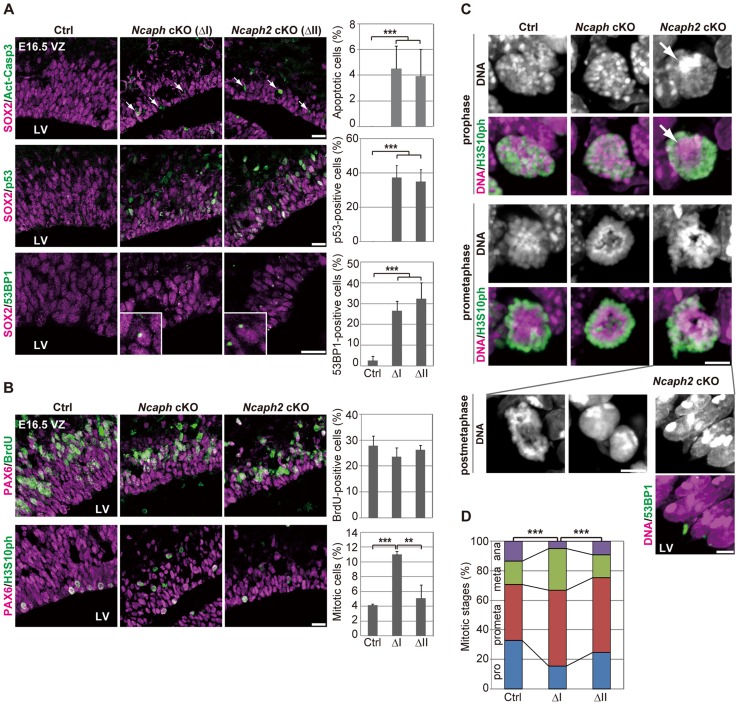
Condensins I and II differentially contribute to NSC divisions and survival. (**A**) Frozen sections of embryonic brains at E16.5 were immunolabeled with antibodies against SOX2 and Act-Casp3 (upper panels), antibodies against SOX2 and p53 (middle panels) or antibodies against SOX2 and 53BP1 (lower panels). The insets show close-ups of 53BP1-positive cells observed in cKO mice. The data shown are from a single representative experiment out of three repeats. Sections from three different embryos of each genotype were analyzed. Bars, 20 µm. The percentages of positive cells in the VZ were measured in the dorsal area of cortices at E16.5, and plotted in the right. Data were obtained from four independent sections from two different embryos. The bars indicate the mean and SD. *** *P*<0.001 (*t*-test with a Holm correction for multiple comparisons). (**B**) One hour after injecting mice at E16.5 with BrdU, frozen sections were prepared and immunolabeled with antibodies against BrdU and PAX6, an NSC marker (upper panels). Alternatively, NSCs in mitosis were visualized by H3S10ph labeling (lower panels). The data shown are from a single representative experiment out of three repeats. Sections from two different embryos of each genotype were used for BrdU incorporation assay and from three different embryos of each genotype were labeled with H3S10ph antibody. Bar, 20 µm. The percentages of BrdU-positive or mitotic cells in the PAX6-positive population were measured and plotted in the right. All data were obtained from three independent sections. The bars indicate the mean and SD. ** *P*<0.01, *** *P*<0.001 (*t*-test with a Holm correction for multiple comparisons). (**C**) Prophase and prometaphase chromosomes on the apical surface of the VZ were immunolabeled with H3S10ph antibody, and their morphologies were analyzed by Hoechst staining. A large Hoechst-dense structure observed in *Ncaph2* cKO mice at prophase is shown by the arrow. Chromatin bridges in anaphase or postmitotic cells were observed only in *Ncaph2* cKO mice (postmetaphase panels). Tail-like chromatin structures in postmitotic cells were immunolabeled with an antibody against 53BP1. The data shown are from a single representative experiment out of three repeats. Sections from three different embryos of each genotype were analyzed. Maximum intensity projections are shown. Bars, 5 µm. (**D**) Mitotic stages were determined based on chromosome morphologies, and their distributions were plotted. Data were obtained from two different embryos (at least 98 mitotic chromosomes scored). *** *P*<0.001 (Chi-squared test).

As mentioned above, *Ncaph* cKO and *Ncaph2* cKO mice exhibited much milder phenotypes than DcKO and *Smc2* cKO mice at E13.5. To elucidate potential functional differences between condensins I and II, we carefully compared the defective phenotypes observed in *Ncaph* cKO and *Ncaph2* cKO mice at E16.5 when targeted subunits were almost completely depleted (S1D and S2D Figures). In both mice, NSCs underwent apoptosis in the VZ, the frequency of which was almost indistinguishable from each other (Fig. 4A, upper panels). Like *Smc2* cKO mice at E13.5, both *Ncaph* cKO and *Ncaph2* cKO mice at E16.5 exhibited nuclear accumulation of p53 (Fig. 4A, middle panels) and focus formation of 53BP1 (Fig. 4A, lower panels) in the VZ. The percentages of the cells with nuclear p53 or 53BP1 foci were similar between the two cKO mice.

To further address the functional differences between condensins I and II, we examined cell cycle progression of NSCs in *Ncaph* cKO and *Ncaph2* cKO mice. BrdU incorporation assay revealed that the frequencies of BrdU incorporation were indistinguishable from control mice (Fig. 4B, upper panels), suggesting that depletion of each condensin does not largely affect S phase progression. On the other hand, the frequencies of mitotic cells, as judged by H3S10ph labeling, were significantly increased in *Ncaph* cKO mice but not *Ncaph2* cKO mice (Fig. 4B, lower panels). These results suggest that loss of condensin I in NSCs slows down mitotic progression, an observation that has also been observed in tissue culture cells [14].

We then focused on mitotic cells observed at E16.5 in *Ncaph* cKO and *Ncaph2* cKO mice, and closely examined their chromosome morphology. The chromosome morphology of condensin I-depleted NSCs was hardly distinguishable from that of control NSCs (Fig. 4C, prophase), although we noticed that an increase in the proportion of prometaphase and metaphase cells (Fig. 4D). Remarkably, condensin II-depleted NSCs displayed unusually large, Hoechst-dense structures at prophase (Fig. 4C, prophase, arrow). Despite the occurrence of such unusual structures, the kinetics of mitotic progression was apparently normal in these cells (Fig. 4D). Morphological defects of chromosomes were also noticed at prometaphase (Fig. 4C, prometaphase) although they were milder than those observed in *Smc2* cKO mice (Fig. 3F). We also detected segregation defects at anaphase and postmitotic cells in *Ncaph2* cKO mice (Fig. 4C, postmetaphase; 0% in control mice, 2.0% in *Ncaph* cKO mice and 62.0% in *Ncaph2* cKO mice; 50 cells were scored per experiment). Moreover, as detected in *Smc2* cKO mice at E13.5, tail-like chromatin structures positive for 53BP1 were observed in the VZ of *Ncaph2* cKO mice. Thus, defects in mitotic chromosome architecture and segregation were observed more prominently in cells depleted of condensin II compared with those of condensin I. Together with the much severer phenotypes observed in *Smc2* cKO mice, we propose that the two condensin complexes have both overlapping and non-overlapping functions in mitotic chromosome dynamics.

### Condensin II prevents hyperclustering of chromocenters in interphase NSCs

**Figure 5:**
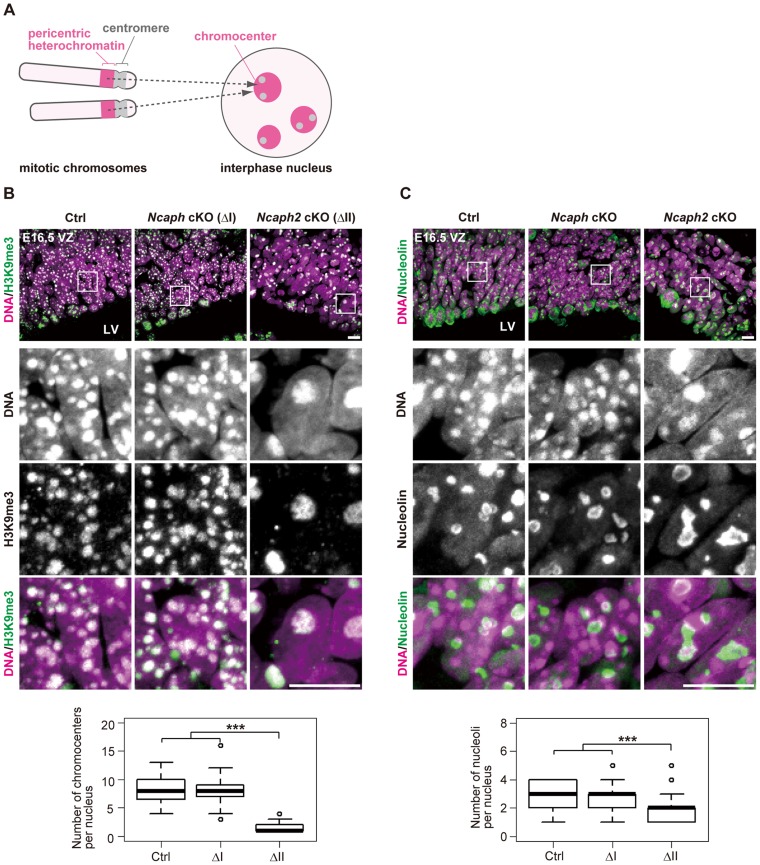
Condensin II prevents hyperclustering of chromocenters in the interphase nucleus in NSCs. (**A**) Shown here is a schematic view of *M. musculus* chromosomes and an interphase nucleus. Pericentric heterochromatin derived from non-homologous chromosomes associate with each other to form clusters within the interphase nucleus. These structures are referred to as chromocenters. (**B**) Frozen sections of embryonic brains at E16.5 were immunolabeled with an antibody against histone H3 trimethylated at lysine 9 (H3K9me3) along with Hoechst staining. Shown on the top are wide areas containing the VZ. The lower panels indicate close-up views of the nuclei indicated by the white rectangles. The data shown are from a single representative experiment out of three repeats. Sections from three different embryos of each genotype were analyzed. Maximum intensity projections are shown. Bars, 10 µm. The numbers of chromocenters per nucleus in the VZ were measured and plotted. Data were obtained from 50 nuclei in the VZ. *** *P*<0.001 (*t*-test with a Holm correction for multiple comparisons). (**C**) Frozen sections of embryonic brains at E16.5 were immunolabeled with an antibody against nucleolin along with Hoechst staining. Wide and close-up views are shown as in (**B**). The data shown are from a single representative experiment out of three repeats. Sections from three different embryos of each genotype were analyzed. Maximum intensity projections are shown. Bars, 10 µm. The numbers of nucleoli per nucleus in the VZ were measured and plotted. Data were obtained analyzed as in (**B**).

What are the Hoechst-dense structures observed in *Ncaph2* cKO mice at prophase? It is known that *Mus musculus* chromosomes contain large blocks of pericentric heterochromatin composed of major satellite repeats. During interphase, the blocks of pericentric heterochromatin from several different chromosomes associate with each other to form a nuclear structure called chromocenters [29] (Fig. 5A). We considered that the Hoechst-dense structures found enlarged in *Ncaph2* cKO mice correspond to chromocenters, as they were labeled with an antibody against histone H3 trimethylated at lysine 9 (H3K9me3)(Fig. 5B). The numbers of chromocenters per nucleus in the VZ were greatly decreased in *Ncaph2* cKO mice, but not in *Ncaph* cKO mice. It seemed reasonable to speculate that the decreased numbers of chromocenters in *Ncaph2* cKO mice would result from hyperclustering of chromocenters [30], [31], rather than loss of chromocenters that is, for example, observed in zygotes [29].

In *M. musculus*, rDNA repeats are present on several chromosomes (known as NORs [nucleolar organizing regions]) and form nucleoli within the interphase nucleus [32]. We found that the structure of nucleoli, as judged by labeling with an anti-nucleolin antibody, was largely disorganized in *Ncaph2* cKO mice, but not in *Ncaph* cKO mice (Fig. 5C). Again, the number of nucleoli per nucleus in both the VZ was decreased in *Ncaph2* cKO mice. Thus, depletion of condensin II in NSCs caused defects in interphase nuclear architecture in NSCs. Very importantly, such defects were hardly observed in cells depleted of condensin I.

### Depletion of condensin II affects nuclear architecture and cell survival in postmitotic neurons

**Figure 6:**
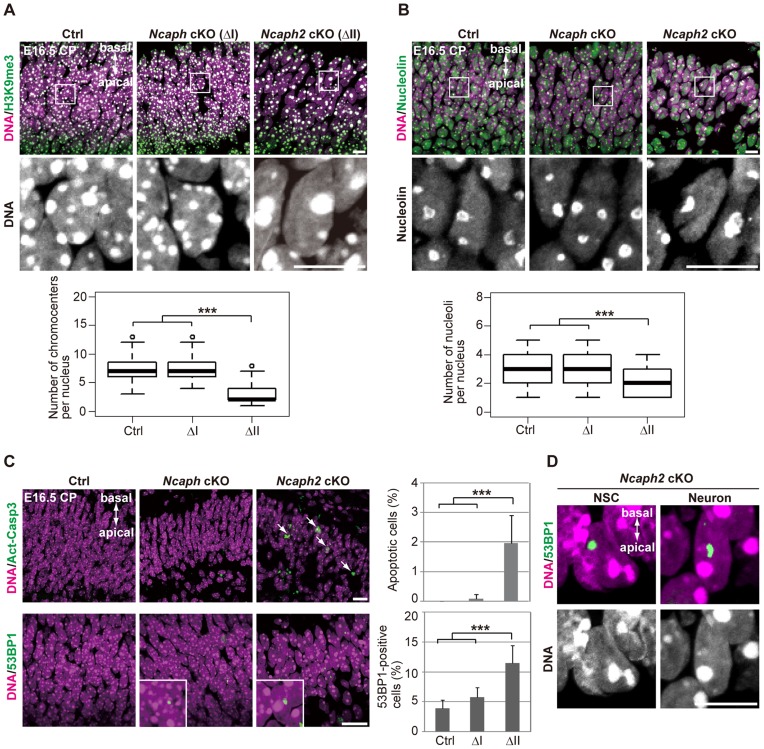
Depletion of condensin II affects nuclear architecture and cell survival in postmitotic neurons. (**A**) Frozen sections of embryonic brains at E16.5 were immunolabeled with an antibody against histone H3 trimethylated at lysine 9 (H3K9me3) along with Hoechst staining. Wide areas containing the CPs and close-up views of the nuclei indicated by the white rectangles are shown. The data shown are from a single representative experiment out of three repeats. Sections from three different embryos of each genotype were analyzed. Maximum intensity projections are shown. Bars, 10 µm. The numbers of chromocenters per nucleus in the CP were measured and plotted. Data were obtained from 100 nuclei in the CP. *** *P*<0.001 (*t*-test with a Holm correction for multiple comparisons). (**B**) The same sections were immunolabeled with an antibody against nucleolin along with Hoechst staining. Wide areas containing the CPs and close-up views of the nuclei indicated by the white rectangles are shown. The data shown are from a single representative experiment out of three repeats. Sections from three different embryos of each genotype were analyzed. Maximum intensity projections are shown. Bars, 10 µm. The numbers of nucleoli per nucleus in the CP were measured and plotted. Data were obtained from 50 nuclei and analyzed as in (**A**). (**C**) Frozen sections of embryonic brains at E16.5 were immunolabeled with antibodies against Act-Casp3 and 53BP1 along with Hoechst staining. The data shown are from a single representative experiment out of three repeats. Sections from three different embryos of each genotype were analyzed. Bars, 20 µm. The numbers of apoptotic and 53BP1-positive cells were measured and plotted. Data were obtained from four independent sections from two different embryos. The bars indicate the mean and SD. *** *P*<0.001 (*t*-test with a Holm correction for multiple comparisons). (**D**) The same sections were immunolabeled with an antibody against 53BP1 along with Hoechst staining. The data shown are from a single representative experiment out of three repeats. Sections from three different embryos of each genotype were analyzed. Maximum intensity projections are shown. Bar, 10 µm.

Remarkably, we noticed that hyperclustering of chromocenters and decreased numbers of nucleoli occurred not only in NSCs but also in postmitoitc neurons in *Ncaph2* cKO mice (Fig. 6A and 6B). Apoptotic cells were detectable in the CP of *Ncaph2* cKO mice, but they were rarely observed in the CP of *Ncaph* cKO mice (Fig. 6C, upper panels). Furthermore, a high incidence of 53BP1-positive cells was observed in the CP of *Ncaph2* cKO mice (Fig. 6C, lower panels). Note that 53BP1 foci did not necessarily colocalize with hyperclustered chromocenters in neurons as well as in NSCs (Fig. 6D), ruling out the possibility that hyperclustering directly causes DNA damage or vice versa. Intriguingly, we nevertheless failed to detect nuclear accumulation of p53 in the CP of *Ncaph2* cKO mice, indicating that a p53-independent mechanism might operate to induce apoptosis in neurons in these mice [33], [34]. Finally, similar hyperclustering of chromocenters in neurons was detectable in DcKO and *Smc2* cKO mice (S6 Figure), further confirming that this defective phenotype was specifically associated with loss of condensin II. These data raise the possibility that depletion of condensin II in NSCs contributes to nuclear architecture in neurons and indirectly affects their survival.

### The relative contribution of condensins I and II to proliferation is cell type-dependent

To what extent can we extend the results obtained with NSCs and neurons to other cell types? To address this question, we used a human retinal pigment epithelial cell line (RPE-1) in the following experiments, which retains non-transformed characters including a normal karyotype. siRNA-treatment efficiently decreased the amounts of condensin subunits (CAP-G [I], CAP-G2 [II] and SMC2 [I and II]) (S7A Figure). 2 d after siRNA treatments, we found that S phase populations largely diminished in cells depleted of CAP-G2 or SMC2, but not in cells depleted of CAP-G (S7B Figure, upper panels). Conversely, cell populations with nuclear p53, p21 and γH2A.X greatly increased in condensin II-deficient cells, but not in condensin I-deficient cells (S7B Figure, middle and lower panels). The tail-like structures connecting two daughter nuclei with DNA damage were observed frequently in cells depleted of CAP-G2 or SMC2 (S7C Figure). When the same set of cells was subjected to a clonogenic survival assay after 9 d, cells depleted of CAP-G2 or SMC2 did not give rise to colonies at all, although the individual cells seemed to be still alive, displaying senescence-like phenotypes that included flattened cellular morphology [35] and large nucleoli [36]. In contrast, CAP-G-depleted cells generated visible colonies, albeit to a lesser degree compared to control cells (S7D Figure). Thus, unlike in mouse NSCs, depletion of condensin II caused p21-induced cell-cycle arrest in RPE-1. These results suggest that the relative contribution of the two condensin complexes to proper chromosome segregation and cell cycle progression is context-dependent, substantially varying among different cell types as well as different organisms (see Discussion).

## Discussion

**Figure 7:**
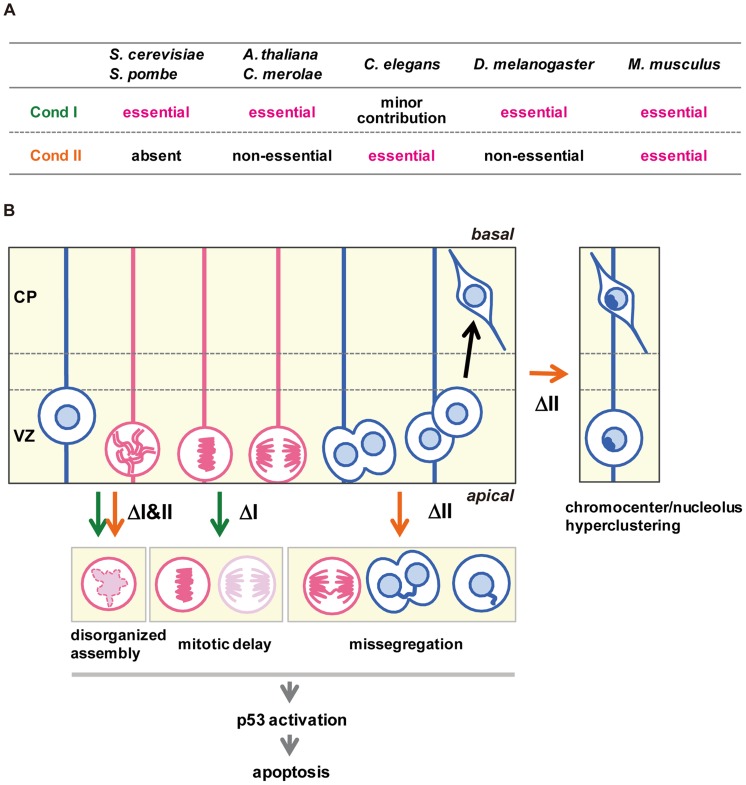
Functional diversity of condensins I and II in evolution and during brain development. (**A**) Requirements of condensins I and II for mitotic cell divisions differ among different model organisms. (**B**) Summary of defective phenotypes observed in NSCs depleted of condensin I, condensin II or both. See the text for details.

The current study represents the first comprehensive genetic study of condensins in mammals. Unlike many other model organisms, our results show that condensins I and II are both essential for early embryonic development as well as cortical development in mice (Fig. 7A). As judged by the anatomical and marker expression analyses, condensins I and II have almost equal contributions to NSC division and subsequent cell survival. Simultaneous depletion of both condensins causes much severer defects than individual depletions, indicating that the two complexes share overlapping functions. Consistent with the anatomical analyses, *Smc2* cKO mice displayed severe defects in chromosome assembly and segregation (Fig. 7B). Furthermore, the unique cell-division patterns during cortical development have allowed us to follow the fate of cells depleted of condensins in the brain. Our results suggest that the severe segregation defect observed in *Smc2* cKO mice causes DNA damage and p53-induced apoptosis, resulting in a failure to build the cerebral cortex.

Although the defective phenotypes observed in *Ncaph* and *Ncaph2* cKO mice were milder than those observed in *Smc2* cKO or DcKO mice, there are notable phenotypic differences between *Ncaph* and *Ncaph2* cKO mice, indicating that condensins I and II have non-overlapping functions, too (Fig. 7B). Chromosome missegregation was observed more prominently in NSCs depleted of condensin II, whereas mitotic delays were detectable only in condensin I-depleted NSCs. This observation raises the possibility that depletion of condensin I might confer an additional source of DNA damage. In fact, DNA damage induced by mitotic delay or arrest has been discussed in the literature although its underlying mechanism remains under debate [37]–[39].

Besides the mitotic defects which result in apoptosis in the VZ, the most dramatic differences between *Ncaph* and *Ncaph2* cKO mice were observed in interphase nuclear architecture: hyperclustering of chromocenters and structural alternation of the nucleolus were uniquely observed in cells depleted of condensin II. Consistently, condensin II, but not condensin I, resides in the nucleus during interphase in NSCs. It is reasonable to speculate that condensin II separates non-homologous chromosomes from each other in NSCs by using the same mechanism that promotes the formation of chromosome territory in *D. melanogaster* polyploid cells [40] or resolves sister chromatids during S phase in human cells [41]. Our results also show that, once nuclear architecture is established by condensin II in NSCs, it seems to be taken over stably to postmitotic neurons. Interestingly, DNA damage and apoptosis were observed in a subpopulation of neurons in *Ncaph2* cKO mice, the mechanism of which is currently unknown. In this sense, it is noteworthy that a hypomorphic mutation of *Ncaph2* was reported to specifically affect T-cell differentiation and survival in mice [42], presumably through a failure in condensin II-dependent structural reorganization of interphase chromatin [43]. On the other hand, it has been shown that overexpression of methyl-CpG-binding protein 2 (MeCP2), whose mutations cause the neurodevelopmental disorder Rett syndrome [44], induce hyperclustering of chromocenters in mouse cells [30], [31]. Moreover, loss of MeCP2 results in structural alternation of the nucleolus in cortical neurons [45], raising the possibility that MeCP2 somehow counteracts condensin II in neurons. Unlike condensin II that functions in NSCs, however, MeCP2 is expressed and functions in postmitotic neurons but not in NSCs [46]. It is therefore reasonable to speculate that condensin II regulates interphase nuclear architecture and neuronal survival independently of MeCP2.

Interestingly, we notice that the relative contribution of the two condensin complexes to cell divisions as well as the downstream responses to condensin depletions is cell type-dependent. In human RPE-1 cells, for instance, depletion of condensin II causes DNA damage and p53 nuclear accumulation, which in turn induces cell cycle arrest through up-regulation of p21. Because DNA damage is far less prominent in condensin I-depleted RPE-1 cells, the proliferative ability of the two sets of cells was strikingly different from each other. Similar yet non-identical results have been reported from a previous study using chicken DT40 cell lines in which individual, specific subunits of condensins I and II were genetically depleted [15]. They showed that depletion of condensin II causes severe segregation defects whereas depletion of condensin I results in a failure in cytokinesis without delaying mitotic progression. On the other hand, a study using mouse embryonic stem (ES) cells reported that condensins I and II have only overlapping functions in cell survival [28]. These studies further support our idea that cellular responses to depletion of condensins are seemingly different among different cell types or cell lines. Taken together, we argue that comparative analyses of the two condensin complexes offer an excellent example of how deep understanding of the physiological complexity requires broad perspectives of cell, developmental and evolutionary biology.

NSCs with oncogenic mutations acquire a high proliferative capacity with impaired differentiation potential, generating malignant brain tumors known as glioblastoma [47], [48]. The mechanistic basis behind tumorigenesis is not fully understood, however. It has recently been reported that SMC2 is overexpressed in colorectal cancer [49] or neuroblastoma [50] cells. Given our results discussed above, it is possible that intricate balancing acts of condensins I and II, rather than simple upregulation or downregulation of their components, are altered in different types of cancer cells. We anticipate that further extension of the current study will help understand the fundamental differences between stem cells and cancer cells, if any, from a point of view of chromosome architecture and dynamics.

## Materials and Methods

### Ethics statement

All animal experiments were carried out in compliance with institutional regulations and were approved by the Director of RIKEN Wako Institute, following a review by the Wako Animal Experiment Committee.

### Generation of condensin cKO mice

Targeted ES cells were generated by using standard conditional knockout methods (inGenious Targeting Laboratories). The structures of targeting vectors are shown in S1, S2, S3 Figures. Chimera mice were produced from these cells and mated with C57BL/6J to establish *Ncaph^Neo/+^*, *Ncaph2^Neo/+^* and *Smc2^Neo/+^* mice. These mice were crossed with *CAG-FLPe* transgenic mice [51] (gift from Dr. Itohara) to delete the *Neo* selection cassettes, resulting in *Ncaph^flox/+^*, *Ncaph2^flox/+^* and *Smc2^flox/+^* mice. Otherwise, the mice with the *Neo* allele were mated with *CAG-Cre* transgenic mice [52] (gift from Dr. Miyazaki) to delete floxed regions, resulting in *Ncaph^Δ/+^*, *Ncaph2^Δ/+^* and *Smc2^Δ/+^* mice. The mice with deletion alleles were further crossed with *NesCre* transgenic mice [18] (Jackson Laboratories, stock #003771). The mutant mice with *flox* alleles or deletion alleles were then backcrossed with C57BL/6J (Japan SLC). Finally, mice analyzed in this study were obtained by mating *Ncaph^Δ/+^* (*Ncaph2^Δ/+^* or *Smc2^Δ/+^*) mice in the *NesCre* background with *Ncaph^flox/flox^* (*Ncaph2^flox/flox^* or *Smc2^flox/flox^*) mice. DcKO mice were generated by mating *Ncaph^Δ/+^*; *Ncaph2^Δ/+^* mice in the *NesCre* background with *Ncaph^flox/flox^*; *Ncaph2^flox/flox^* mice. Noon of the day when a vaginal plug was observed was defined as embryonic day 0.5 (E0.5). Genomic structures of the mutant mice were confirmed by Southern blot analysis (S1, S2, S3 Figures) with DIG-labeled probes generated by using PCR DIG Probe Synthesis Kit (Roche). Primer sequences for probe synthesis are shown in S1 Table. Hybridization and detection of hybridization signals were performed as described previously [48]. For genotyping of the mice, genomic DNA was isolated from the tail by a standard method and then used for PCR with specific primers (S1 Table). Cycle parameters were 20 sec at 94°C, 60 sec at 60°C, and 60 sec at 72°C for 30 cycles.

### Cell cultures

NSCs were prepared from the cerebral cortex at E13.5 or E16.5 and cultured in DMEM/F12 (Life Technologies) supplemented with bFGF (10 ng/ml, Peprotech), EGF (10 ng/ml, Peprotech), Heparin (5 µg/ml, Sigma) and B27 (Life Technologies). The cells were maintained as neurospheres. For adherent monolayer cultures, NSCs were cultured in dishes (or on coverslips) coated with poly-D-lysine (50 µg/ml, Sigma) and fibronectin (1 µg/ml, Sigma). RPE-1 cells (ATCC CRL-4000) were cultured on dishes or poly-D-lysine-coated coverslips in DMEM (Life Technologies) supplemented with 10% FBS (Cell Culture Biosciences).

### Immunohistochemical analysis

Whole embryos or tissues dissected were fixed with 4% paraformaldehyde (PFA) in PBS at 4°C for 24 h, cryoprotected in 12–18% sucrose, frozen in O.C.T. Compound (Sakura Finetek) by soaking in liquid N_2_ and kept at −80°C until use. Frozen blocks were serially sectioned at a thickness of 10 µm by using the cryostat CM3050S (Leica), and retrieved on MAS-coated slides (Matsunami). Dorsal parts of the developing cortex in coronal sections were selected for analysis. All slides were heated at 90°C for 20 min in citrate buffer (HistoVT One, Nacalai) to retrieve antigens, washed three times with PBS, followed by incubation with blocking buffer (2% skim milk and 0.3% Triton X-100 in PBS). The sections on the slides were incubated with primary antibodies at appropriate dilutions in blocking buffer at 4°C overnight. After washed three times for 10 min with PBS, the sections were incubated with secondary antibodies at room temperature for 1 h. The sections were then counterstained with Hoechst 34580 (2 µg/ml, Life Technologies), washed twice for 10 min with PBS, and mounted in a mounting medium (Fluorescent Mounting Medium, DAKO). For bromodeoxyuridine (BrdU, Sigma) labeling, pregnant mice were intraperitoneally injected with 500 µl of PBS containing 1 mg of BrdU. Mice were sacrificed 1 h after injection, and embryos were processed for cryosections as described above. All images were obtained by using an LSM710 confocal microscope and ZEN2010 software (Carl Zeiss) and handled with Photoshop CS4 (Adobe). Antibodies used are listed in S2 Table.

### Immunocytochemical analysis

Cells grown on coverslips were fixed with 2% PFA in PBS for 30 min, permeabilized with 0.5% Triton X-100 in PBS for 5 min, and washed three times with PBS at room temperature. The cells were incubated with 3% BSA in PBS for 30 min, and then incubated with primary antibodies for 1 h. After washed twice for 5 min with PBS, the cells were treated with fluorescently labeled secondary antibodies at room temperature for 1 h. The labeled cells were counterstained with Hoechst 34580 (2 µg/ml) for 15 min, washed twice for 10 min with PBS, and finally mounted in the mounting medium. For immunolabeling of metaphase chromosome spreads, NSCs were cultured with colcemid (0.05 µg/ml, Life Technologies) for 3 h before harvesting, treated with 75 mM KCl at 37°C for 20 min, and centrifuged onto coverslips at 800 rpm for 2 min in a cytocentrifuge (Cytospin 4, Thermo Shandon). The cytospun preparations were fixed and immunolabeled as described above. For 5-ethynyl-2′-deoxyuridine (EdU, Life Technologies) labeling, RPE-1 cells were cultured with 10 µM EdU for 1 h before fixation. The EdU incorporated was detected with the Click-iT EdU Imaging Kits according to the manufacturer's instructions (Life Technologies). Images were obtained by using LSM710. Antibodies used are listed in S2 Table.

### TUNEL assay

Terminal deoxynucleotidyltransferase-mediated dUTP-biotin nick end labeling (TUNEL) assays were performed according to the manufacturer's instruction (In Situ Cell Death Detection Kit, Roche). In brief, cryosections were fixed with 4% PFA in PBS for 20 min, washed with PBS for 30 min, permeabilized with citrate buffer containing 0.03% Triton X-100, and incubated with TUNEL reaction solution at 37°C for 1 h. The sections were counterstained with Hoechst 34580 (2 µg/ml) for 15 min, washed twice for 10 min with PBS, and mounted in the mounting medium. Images were obtained by using LSM710.

### Hematoxylin and eosin staining

Cryosections were washed three times with PBS and soaked in Mayer's hematoxylin solution (Wako) for 5 min. The slides were washed with water for 5 min, transferred into eosin-Y solution (Wako) for 20 sec, washed twice with water and mounted in the mounting medium. Images were obtained by using LSM710.

### Image acquisition

Confocal images were acquired with an LSM710 equipped with an Axio Observer.Z1 inverted microscope that was regulated with ZEN2010 software (Carl Zeiss). Images were collected as 2 µm-thick optical sections with ×10 (0.3 NA, Plan-Neofuar) or ×20 (0.8 NA, Plan Apochromat) objectives, or 1 µm-thick optical sections with ×63 (1.4 NA, Plan Apochromat) or ×100 objectives (1.46 NA, Plan Apo) with oil immersion. For acquisition of the data from mitotic chromosomes and nuclear architecture, Z-stack images were obtained as 5–10 µm thickness and processed into maximum intensity projection images. All imaging data were converted into TIFF format and handled with Photoshop CS4 (Adobe).

### Preparation of extracts from brains and NSCs

Dissected brains from mouse embryos at E12.5 were transferred into extraction buffer (20 mM HEPES-KOH, pH 8.0, 100 mM KCl, 2 mM MgCl_2_ and 10% glycerol) supplemented with a protease inhibitor cocktail tablet (Roche). The brains were homogenized, sonicated, and centrifuged at 20,000 g for 30 min at 4°C. The supernatants were taken and used for western blot analysis and immunoprecipitation.

### Immunoprecipitation and western blot analysis

Extracts from brains were incubated with specific antibodies at 4°C for 1 h with rotor agitation (S2 Table). The mixtures were transferred into fresh microtubes containing slurry of rProtein A Sepharose (GE Healthcare) and incubated for another 1 h. The beads were then washed five times with extraction buffer, and the immunoprecipitates were subjected to western blot analysis. For western blot analysis of extracts or immunoprecipitates, an equal volume of 2× SDS sample buffer (125 mM Tris-HCl, pH 6.8, 2% SDS, 20% glycerol, 100 mM DTT, and bromophenol blue) was mixed with samples and boiled for 3 min. For analysis of total lysates, cells or dissociated brains were suspended in extraction buffer (2×10^7^ cells/ml), followed by the addition of an equal volume of 2× SDS sample buffer and boiled for 3 min. Protein samples were subjected to SDS-PAGE and transferred to nitrocellulose membranes (Hybond-ECL, GE Healthcare). The protein-bound membranes were incubated with blocking buffer (5% skim milk, 0.05% tween 20, 1× TBS) for 30 min, and then incubated with primary antibodies diluted in the same buffer for 1 h. The membranes were washed three times for 10 min with TBST (0.05% Tween 20, 1× TBS), incubated with secondary antibodies for 1 h. After washed three times for 10 min with TBST, the membranes were treated with substrates (Immobilon Western Chemilluminescent HRP Substrate, Millipore) for 1 min at room temperature, and the signals were detected by LAS3000 (Fuji Film). Antibodies used are listed in S2 Table.

### RNA isolation, cDNA synthesis and real-time RT-PCR

Total RNA was extracted from developing brains using TRIzol reagent (Life Technologies) according to the manufacturer's protocol. The RNA was treated with TURBO DNase (Life Technologies) at 37°C for 30 min, purified with RNeasy (QIAGEN) to remove genomic DNA, and used as a template to synthesize cDNA using Superscript III (Life Technologies). Real-time RT-PCR was performed using a CFX96 Real-Time PCR Detection System and C1000 Thermal Cycler (Bio-Rad) in a 20-µl reaction mixture containing 0.5 µl cDNA, 0.5 nM specific primers (S1 Table), and 10 µl SsoFast EvaGreen Super Mix (Bio-Rad). mRNA levels of target genes were normalized to the mRNA level of *Gapdh*. Four technical replicates were made and analyzed for each experiment.

### Data analysis

Representative images obtained from at least two independent experiments are shown in each figure. Bar graphs were depicted with Excel software (Microsoft) and box plots were with R software (http://www.r-project.org/). The statistical significance in mean values of two-sample comparison was determined with Welch's *t*-test. For the comparison of multiple sample sets, *t*-test with a Holm correction after one-way ANOVA test was performed. Chi-squared test was applied to compare the difference of proportions or Mendelian expectations. *P* values from all data sets were calculated with R software.

### siRNA-mediated depletions of condensin subunits

RPE-1 cells were seeded on 6-well culture plates and incubated overnight. The cells were then treated with siRNA duplexes at a final concentration of 32 nM using Lipofectamine RNAiMAX (Life Technologies) for 6 h. For efficient depletion, siRNA treatments were performed two cycles (once a day). The sequences of the Stealth siRNAs used were as follows: hSMC2, 5′-CUUCAAUGCUAUCACUGGCUUAAAU-3′; hCAP-G, 5′-CUUAAAGUCUCAUGAAGCAAACAGC-3′; hCAP-G2, 5′-AGCCCUACUGGAAUGUGUUAUUAUA-3′. Negative Control Medium GC Duplexes (Life Technologies) was used as a negative control.

### Clonogenic survival assay

RPE-1 cells treated with siRNA were plated into dishes (3,500 cells per 60 cm^2^) and cultured for 9 d. The cells were fixed with methanol for 1 min and subsequently stained with 4% Giemsa solution (Roche) for 15 min. After washed with water, the stained colonies were dried and observed. Images were obtained by using a digital camera (Leica, D-LUX3) and handled with Photoshop CS4 (Adobe).

## Supporting Information

S1 Figure Generation of *Ncaph* cKO mice. (**A**) The *Ncaph* locus in wild-type (WT) ES cells was targeted with the vector drawn in this scheme. Homologous recombination resulted in insertion of the *loxP* sites and the Neomycin selection cassette, giving rise to *Ncaph^Neo^*. The selection cassette was then removed by crossing with FLP deleter mice to produce *Ncaph^flox^*. Finally, deletion of the floxed exons was achieved by crossing with mice expressing Cre recombinase. The resulting *Ncaph^Δ2–4^* allele lacks exons 2–4. The numbered white boxes indicate exons. Also shown are positions of the hybridization probe and *Bam*HI sites used for Southern blot analysis. (**B**) Genomic DNA was purified from tail tips of WT and *Ncaph^Neo/+^* mice and digested with *Bam*HI. Southern blot analysis was performed using the *Ncaph* probe. Successful targeting would give rise to an 11.1-kb fragment, which was shorter than the size of WT (15.3 kb). Expected band pattern was indeed observed from *Ncaph^Neo/+^* genomic DNA, indicating correct targeting of the locus. (**C**) Genomic DNA was subjected to PCR analysis using specific primers as shown in (**A**). Expected sizes of PCR products were detected for all genotypes, thereby confirming correct targeting. (**D**) Frozen sections of embryonic brains were immunolabeled with an antibody against CAP-H, and stained with Hoechst. The fluorescent intensity of CAP-H was decreased but still detectable in the VZ at E13.5 (left panels). At E16.5, CAP-H was hardly detectable in the VZ, indicating its almost complete loss in NSCs. The data shown are from a single representative experiment out of three repeats. Sections from three different embryos of each genotype were analyzed. Bar, 50 µm.
(PDF)

S2 Figure Generation of *Ncaph2* cKO mice. (**A**) The *Ncaph2* locus in WT ES cells was targeted with the vector drawn in this scheme. Homologous recombination resulted in insertion of the *loxP* sites and the Neomycin selection cassette, giving rise to *Ncaph*2*^Neo^*. The selection cassette was removed by crossing with FLP deleter mice to produce *Ncaph*2*^flox^*. Finally, deletion of the floxed exons was achieved by crossing with mice expressing Cre recombinase. The resulting *Ncaph2^Δ3–8^* allele lacks exons 3–8. The numbered white boxes indicate exons. Also shown are positions of the hybridization probe and *Bcl*I sites used for Southern blotting analysis. (**B**) Genomic DNA was purified from tail tips of WT and *Ncaph2^Neo/+^* mice and digested with *Bcl*I. Southern blot analysis was performed using the *Ncaph2* probe. Successful targeting would give rise to a 7.3-kb fragment, which was shorter than the size of WT (8.5 kb). Expected band pattern was indeed observed from *Ncaph2^Neo/+^* genomic DNA, indicating correct targeting of the locus. (**C**) Genomic DNA was subjected to PCR analysis using specific primers as shown in (**A**). Expected sizes of PCR products were detected for all genotypes, thereby confirming correct targeting. (**D**) Frozen sections of embryonic brains were immunolabeled with an antibody against CAP-H2, and stained with Hoechst. The fluorescent intensity of CAP-H2 was decreased but still detectable in the VZ at E13.5 (left panels). At E16.5, CAP-H2 was hardly detectable in the VZ, indicating its almost complete loss in NSCs. The data shown are from a single representative experiment out of three repeats. Sections from three different embryos of each genotype were analyzed. Bar, 50 µm.
(PDF)

S3 Figure Generation of *Smc2* cKO mice. (**A**) The *Smc2* locus in WT ES cells was targeted with the vector drawn in this scheme. Homologous recombination resulted in insertion of the *loxP* sites and the Neomycin selection cassette, giving rise to *Smc2^Neo^*. The selection cassette was removed by crossing with FLP deleter mice to produce *Smc2^flox^*. Finally, deletion of the floxed exons was achieved by crossing with mice expressing Cre recombinase. The resulting *Smc2^Δ10–12^* allele lacks exons 10–12. The numbered white boxes indicate exons. Also shown are positions of the hybridization probe and *Mfe*I sites used for Southern blotting. (**B**) Genomic DNA was purified from tail tips of WT and *Smc2^Neo/+^* mice and digested with *Mfe*I. Southern blot analysis was performed using the *Smc2* probe. Successful targeting would give rise to a 10.8-kb fragment, which was shorter than the size of WT (19.1 kb). Expected band pattern was indeed observed from *Smc2^Neo/+^* genomic DNA, indicating correct targeting of the locus. (**C**) Genomic DNA was subjected to PCR analysis using specific primers as shown in (**A**). Expected sizes of PCR products were detected for all genotypes, thereby confirming correct targeting. (**D**) Frozen sections of embryonic brains were immunolabeled with an antibody against SMC2, and stained with Hoechst. The fluorescent intensity of SMC2 was greatly reduced in the VZ at E13.5. The data shown are from a single representative experiment out of three repeats. Sections from three different embryos of each genotype were analyzed. Bar, 50 µm.
(PDF)

S4 Figure Condensins I and II are both essential for early embryonic development and cortical development. (**A**) Conventional knockout mice were generated by crossing conditional knockout mice with transgenic mice expressing Cre recombinase ubiquitously. Heterozygotes bearing deletions were mated with each other, and the uterus of female mice was checked for the presence of homozygotes at E12.5. In a representative uterus shown here, empty deciduae (indicated by the arrows) were observed that would have contained homozygotes. Bar, 10 mm. (**B**) Genotypes of living embryos were determined by PCR analysis. None of the living embryos were judged to be homozygotes (*Δ/Δ*), suggesting that the homozygotes had disappeared after their implantations. *P* value was obtained from Chi-squared test, indicating significant deviation from an expected Mendelian ratio. (**C**) Frozen sections of embryonic brains at the stages indicated were stained with hematoxylin and eosin (H&E). Subtle if any defects were apparent at E13.5 in all mutant mice. By E16.5, however, the brain structures became highly disorganized in DcKO and *Smc2* cKO mice. Although morphological defects were relatively mild in *Ncaph* cKO and *Ncaph2* cKO mice at E16.5, the number of cells in the cortex seemed decreased. By E19.5, disorganized cerebral cortices became apparent in both *Ncaph* cKO and *Ncaph2* cKO mice. The data shown are from a single representative experiment out of two repeats. Sections from two different embryos of each genotype were analyzed. Bar, 200 µm. (**D**) Frozen sections of embryonic brains at E13.5 were subjected to a TUNEL assay to detect apoptotic cell death. DNA was counterstained with Hoechst. Whereas *Ncaph* cKO and *Ncaph*2 cKO mice displayed a mild increase in apoptotic cells, massive apoptosis was detected in DcKO and *Smc2* cKO mice. The data shown are from a single representative experiment out of two repeats. Sections from two different embryos of each genotype were analyzed. Bar, 100 µm.
(PDF)

S5 Figure Condensins I and II ensure NSC proliferation in culture. (**A**) Cells were obtained from the cerebral cortex at E13.5 in control and *Smc2* cKO mice, and cultured *in vitro* for 4 d (4 DIV). The data shown are from a single representative experiment out of three repeats. Bar, 100 µm. (**B**) Neurospheres formed after 4-d culture were classified based on their diameter, and plotted. The insets indicate representative neurospheres with different diameters (µm). Neurospheres from *Smc2* cKO mouse (red) were smaller than those from control mouse (blue). (**C**) The same number of cells from each brain was plated in culture dishes. After allowing neurosphere to form for 3 d (3 DIV), NSCs are dissociated and their numbers were scored. Data were obtained from three independent cultures and normalized to the mean number of NSCs from control mice as 100%. Bars indicate the mean and SD. *** *P*<0.001 (*t*-test). The number of NSCs was dramatically decreased in *Smc2* cKO, suggesting their defects in cell proliferation. (**D**) Cells obtained from the cerebral cortex at E13.5 were cultured for 1 d on coverslips (1 DIV) and immunolabeled with antibodies against PAX6 and γH2A.X. DNA was counterstained with Hoechst. γH2A.X-positive chromatin bridges were observed in PAX6-positive NSCs derived from *Smc2* cKO brains, which was consistent with the data obtained *in vivo*. The data shown are from a single representative experiment out of two repeats. Bar, 20 µm.
(PDF)

S6 Figure Condensin II prevents hyperclustering of chromocenters in neurons, too. (**A**) Frozen sections of embryonic brains at E16.5 were fluorescently labeled with antibodies against subtype-specific markers (TBR1 [for deep-layer neurons] and BRN2 [for upper-layer neurons]). DNA was counterstained with Hoechst. Shown here are images focused on TBR1-positive, deep-layer neurons. Hyperclustered chromocenters in neurons were detected in *Ncaph2* cKO, DcKO and *Smc2* cKO mice, but not in control or *Ncaph* cKO mice. The data shown are from a single representative experiment out of two repeats. Sections from two different embryos of each genotype were analyzed. Maximum intensity projections are shown. Bar, 20 µm. (**B**) The numbers of chromocenters per nucleus were measured in TBR1-positive neurons in the cortical plate and plotted. Data were obtained from 50 nuclei. ** *P*<0.01, *** *P*<0.001 (*t*-test with a Holm correction for multiple comparisons). The numbers of chromocenters per nucleus were significantly decreased in *Ncaph2* cKO, DcKO and *Smc2* cKO mice compared to control and *Ncaph* cKO mice, implicating condensin II-specific functions for properly organizing nuclear architecture.
(PDF)

S7 Figure Cell type-dependent contributions of condensins I and II to cell proliferation. (**A**) RPE-1 cells were treated twice with the siRNAs indicated, plated on culture dishes, and cultured for 1 d. The cells were then harvested and analyzed by western blotting using antibodies against human condensin subunits (CAP-G, CAP-G2 and SMC2) and GAPDH (an internal control). (**B**) The same set of cells treated with the siRNAs was plated on coverslips and cultured for 2 d. The cells were pulse labeled with 5-ethynyl-2′-deoxyuridine (EdU) before fixation (first row). The percentages of EdU-positive cells were measured, and plotted in the right. RPE-1 cells depleted of CAP-G or SMC2 were barely positive for EdU labeling, whereas CAP-G-depleted cells did not show a significant reduction of EdU-positive cells. Alternatively, the same set of the siRNA-treated cells was immunolabeled with antibodies against p53 (second row), p21 (third row) and γH2A.X (forth row). The percentages of cells with nuclear p53 or with p21 or with γH2A.X foci were measured and plotted in the right. Cells depleted of CAP-G2 or SMC2 displayed high levels of p53-positive, p21-positive and γH2A.X-positive cells, suggesting strongly that loss of condensin II causes DNA damage, p53 nuclear accumulation and p21 up-regulation. These results suggest that depletion of condensin II causes p21-induced cell cycle arrest in RPE-1 cells. All data were obtained from six independent fields from two different cultures. The bars indicate the mean and SD. *** *P*<0.001 (*t*-test with a Holm correction for multiple comparisons). The panels shown are from a single representative experiment out of three repeats. Bars, 100 µm. (**C**) γH2A.X-positive chromatin bridges were observed in cells depleted of CAP-G2 or SMC2, but not of CAP-G (not shown). The panels shown are from a single representative experiment out of three repeats. Bars, 10 µm. (**D**) RPE-1 cells were treated twice with the siRNAs indicated, plated on culture dishes, and cultured for 9 d. The dishes were then subjected to Giemsa staining to visualize colonies formed. RPE-1 cells depleted of CAP-G generated a decreased number of colonies compared with control cells (upper panels). On the other hand, cells depleted of CAP-G2 or SMC2 produced no visible colonies, although the cells seemed to be still alive on the dishes and exhibit flattened cellular morphology (lower panels). These data suggest that condensin II plays a crucial role in cell proliferation in this experimental setup. The data shown are from a single representative experiment out of two repeats. Bar, 20 µm.
(PDF)

S1 Table Primer sequences used in this study.
(PDF)

S2 Table Antibodies used in this study.
(PDF)
