# Supplementary figures and images for: Overlapping and Non-overlapping Functions of Condensins I and II in Neural Stem Cell Divisions
Source: PLoS Genet. 2014 Dec 4;10(12):e1004847. doi: 10.1371/journal.pgen.1004847 (PMC4256295; doi:10.1371/journal.pgen.1004847)

**A**

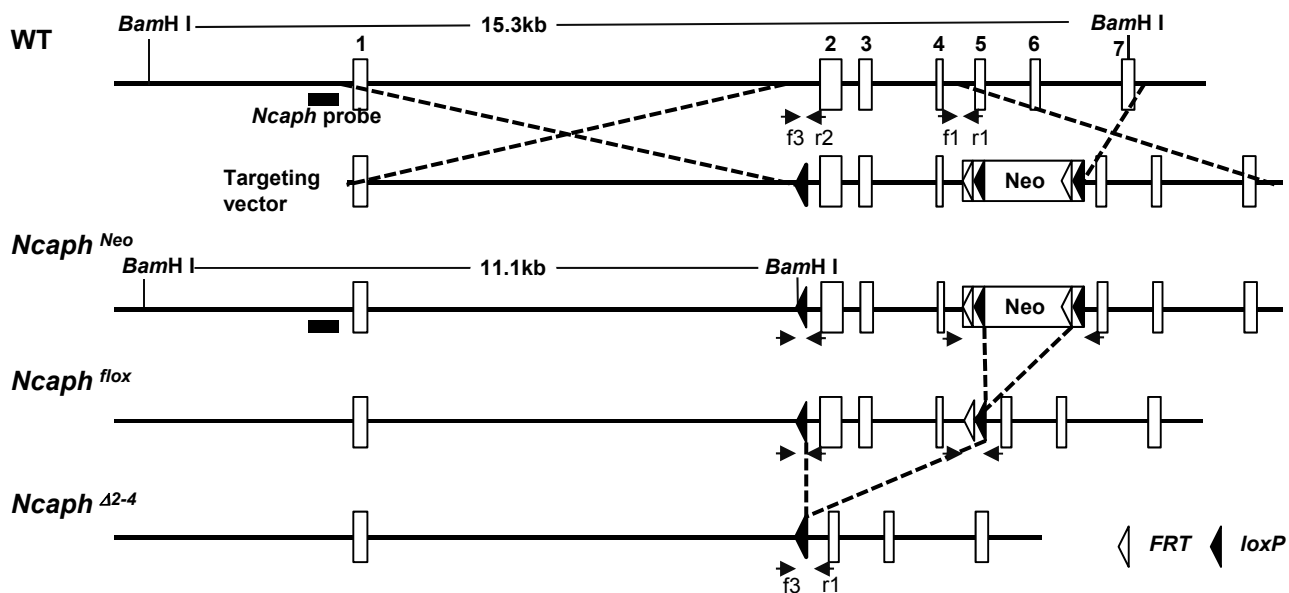

**B**

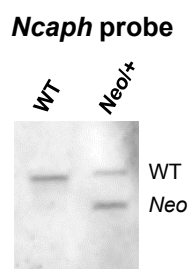

**C**

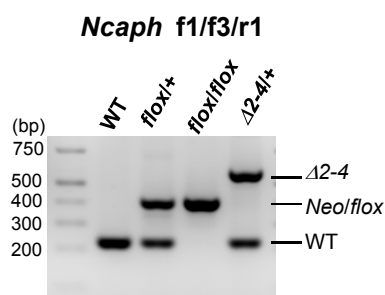

**D**

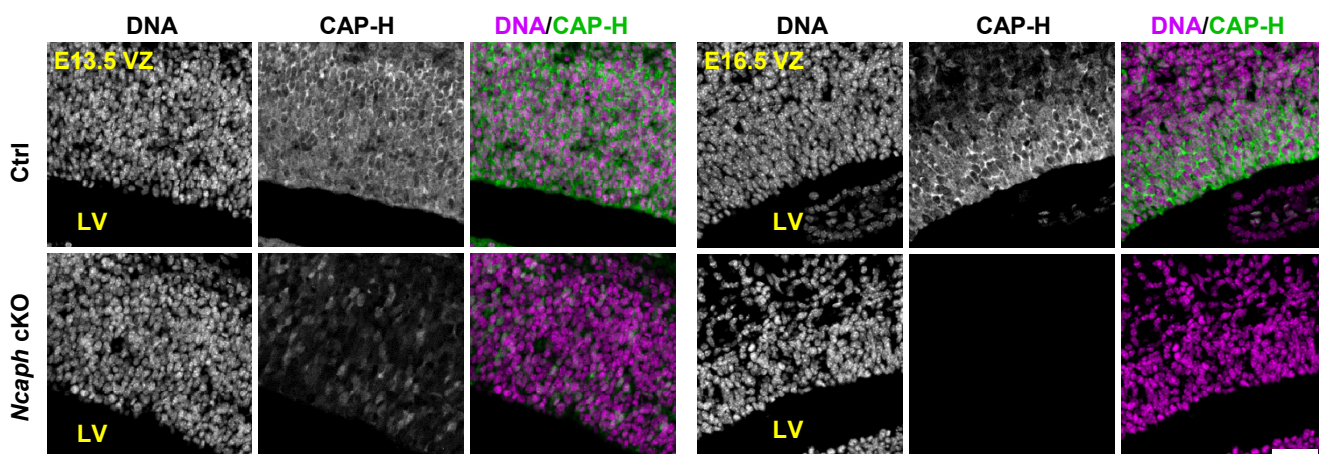

Supplement: S1 Figure — Generation of Ncaph cKO mice. (A) The Ncaph locus in wild-type (WT) ES cells was targeted with the vector drawn in this scheme. Homologous recombination resulted in insertion of the loxP sites and the Neomycin selection cassette, giving rise to NcaphNeo. The selection cassette was then removed by crossing with FLP deleter mice to produce Ncaphflox. Finally, deletion of the floxed exons was achieved by crossing with mice expressing Cre recombinase. The resulting NcaphΔ2–4 allele lacks exons 2–4. The numbered white boxes indicate exons. Also shown are positions of the hybridization probe and BamHI sites used for Southern blot analysis. (B) Genomic DNA was purified from tail tips of WT and NcaphNeo/+ mice and digested with BamHI. Southern blot analysis was performed using the Ncaph probe. Successful targeting would give rise to an 11.1-kb fragment, which was shorter than the size of WT (15.3 kb). Expected band pattern was indeed observed from NcaphNeo/+ genomic DNA, indicating correct targeting of the locus. (C) Genomic DNA was subjected to PCR analysis using specific primers as shown in (A). Expected sizes of PCR products were detected for all genotypes, thereby confirming correct targeting. (D) Frozen sections of embryonic brains were immunolabeled with an antibody against CAP-H, and stained with Hoechst. The fluorescent intensity of CAP-H was decreased but still detectable in the VZ at E13.5 (left panels). At E16.5, CAP-H was hardly detectable in the VZ, indicating its almost complete loss in NSCs. The data shown are from a single representative experiment out of three repeats. Sections from three different embryos of each genotype were analyzed. Bar, 50 µm. (PDF) [file pgen.1004847.s001.pdf]

**A**

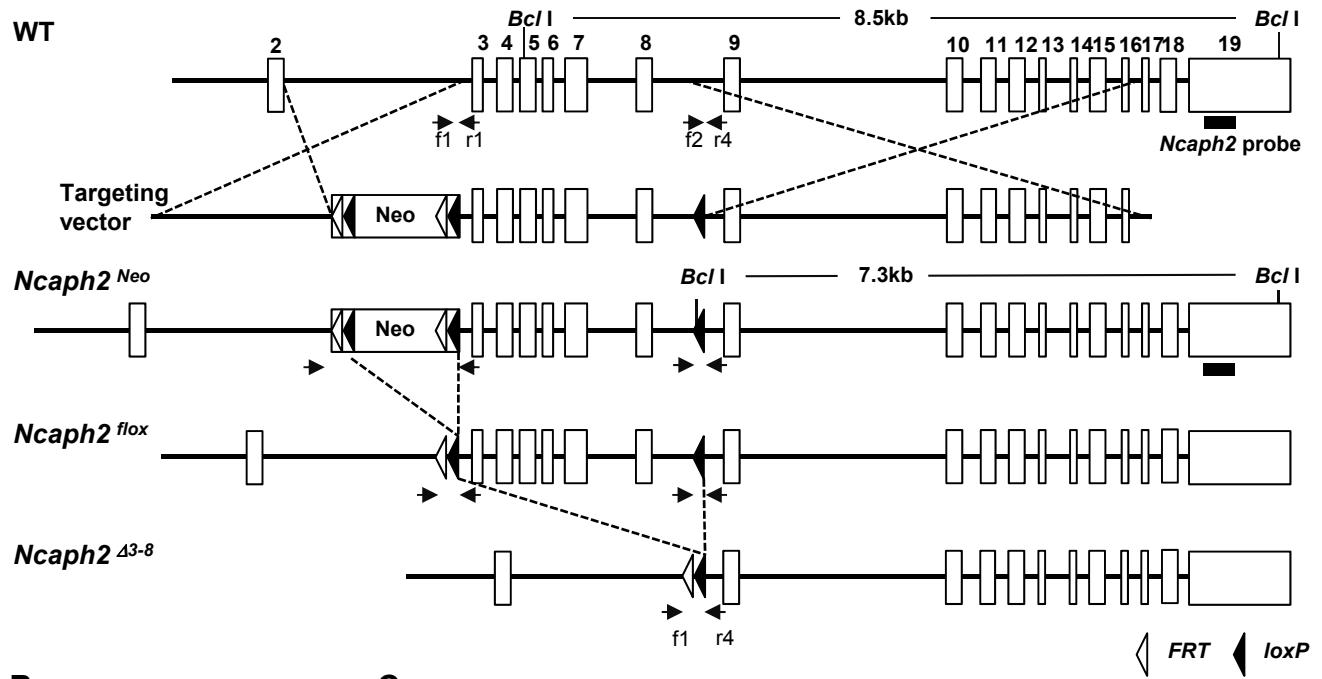

**B**

*NcapH2* probe

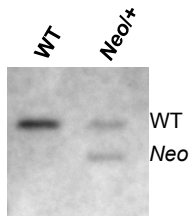

**C**

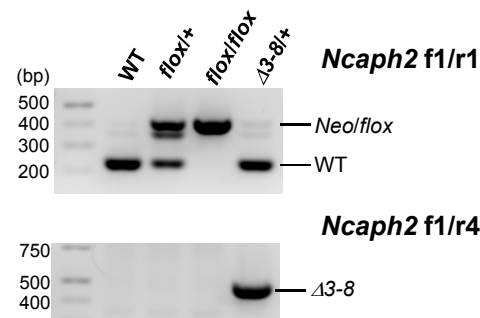

**D**

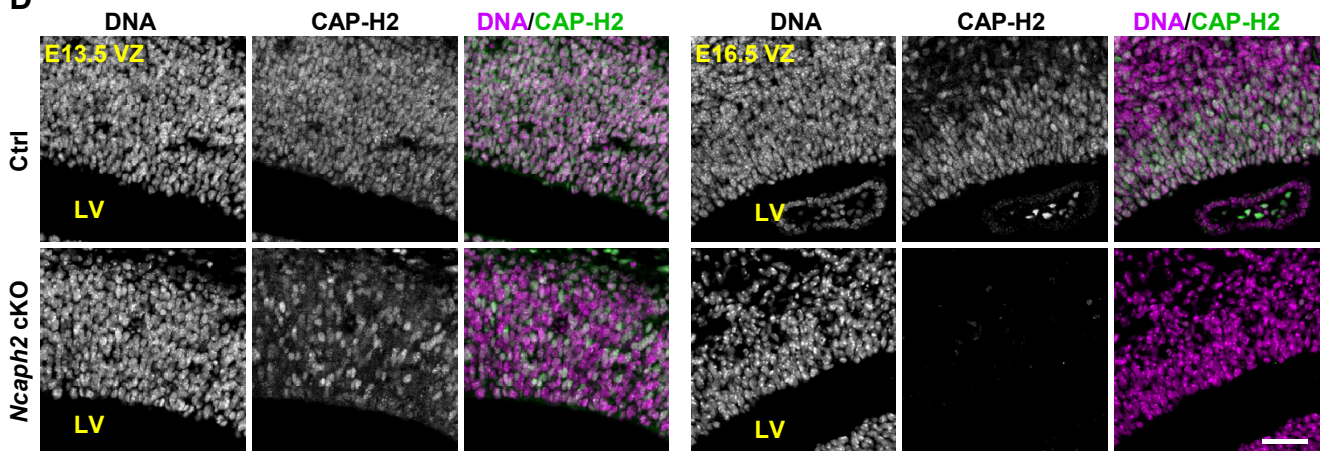

Supplement: S2 Figure — Generation of Ncaph2 cKO mice. (A) The Ncaph2 locus in WT ES cells was targeted with the vector drawn in this scheme. Homologous recombination resulted in insertion of the loxP sites and the Neomycin selection cassette, giving rise to Ncaph2Neo. The selection cassette was removed by crossing with FLP deleter mice to produce Ncaph2flox. Finally, deletion of the floxed exons was achieved by crossing with mice expressing Cre recombinase. The resulting Ncaph2Δ3–8 allele lacks exons 3–8. The numbered white boxes indicate exons. Also shown are positions of the hybridization probe and BclI sites used for Southern blotting analysis. (B) Genomic DNA was purified from tail tips of WT and Ncaph2Neo/+ mice and digested with BclI. Southern blot analysis was performed using the Ncaph2 probe. Successful targeting would give rise to a 7.3-kb fragment, which was shorter than the size of WT (8.5 kb). Expected band pattern was indeed observed from Ncaph2Neo/+ genomic DNA, indicating correct targeting of the locus. (C) Genomic DNA was subjected to PCR analysis using specific primers as shown in (A). Expected sizes of PCR products were detected for all genotypes, thereby confirming correct targeting. (D) Frozen sections of embryonic brains were immunolabeled with an antibody against CAP-H2, and stained with Hoechst. The fluorescent intensity of CAP-H2 was decreased but still detectable in the VZ at E13.5 (left panels). At E16.5, CAP-H2 was hardly detectable in the VZ, indicating its almost complete loss in NSCs. The data shown are from a single representative experiment out of three repeats. Sections from three different embryos of each genotype were analyzed. Bar, 50 µm. (PDF) [file pgen.1004847.s002.pdf]

**A**

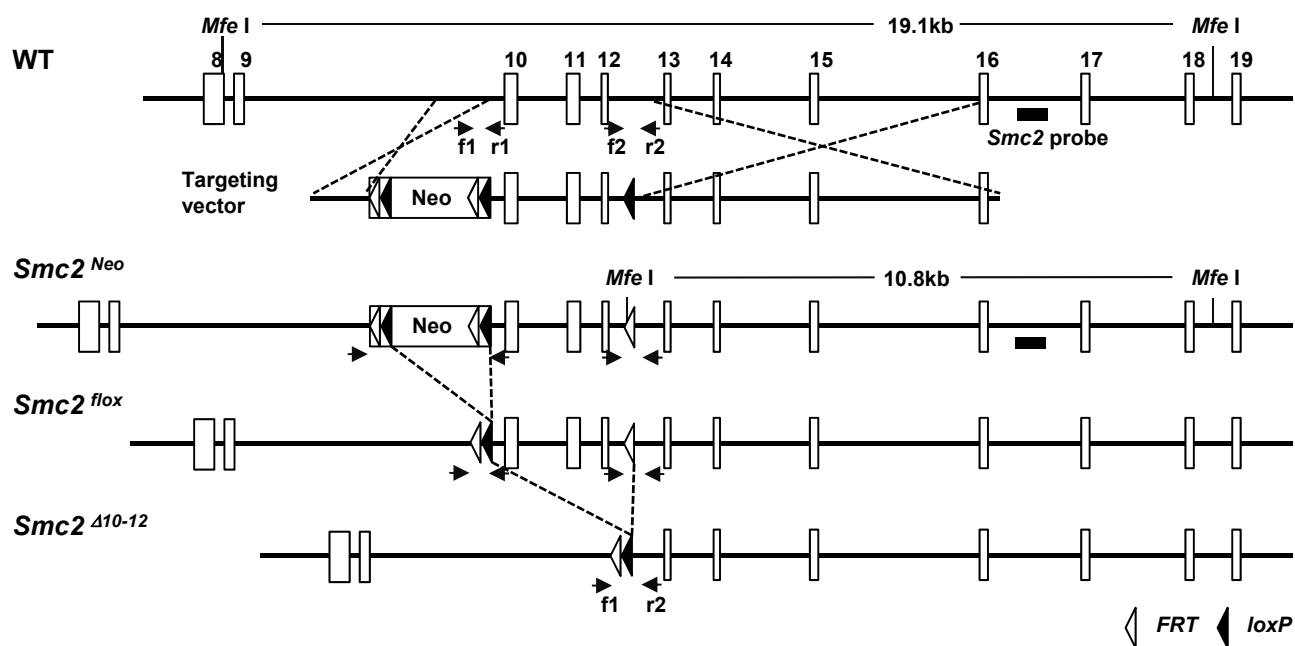

**B**

**Smc2 probe**

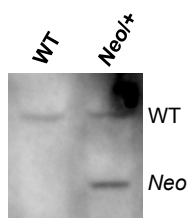

**C**

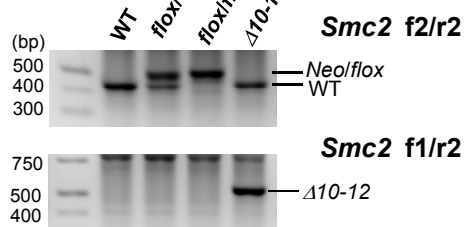

**D**

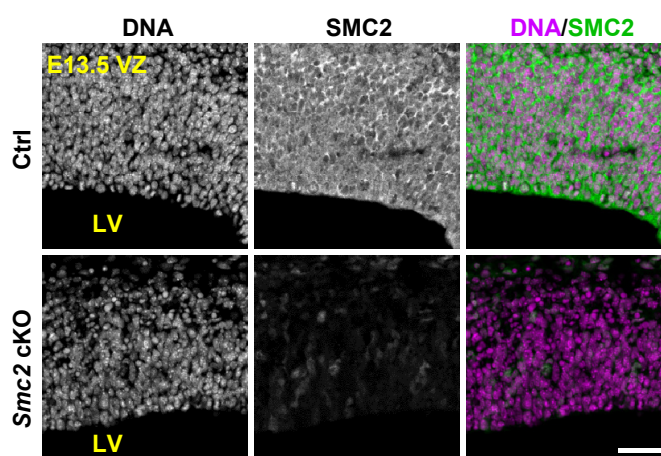

Supplement: S3 Figure — Generation of Smc2 cKO mice. (A) The Smc2 locus in WT ES cells was targeted with the vector drawn in this scheme. Homologous recombination resulted in insertion of the loxP sites and the Neomycin selection cassette, giving rise to Smc2Neo. The selection cassette was removed by crossing with FLP deleter mice to produce Smc2flox. Finally, deletion of the floxed exons was achieved by crossing with mice expressing Cre recombinase. The resulting Smc2Δ10–12 allele lacks exons 10–12. The numbered white boxes indicate exons. Also shown are positions of the hybridization probe and MfeI sites used for Southern blotting. (B) Genomic DNA was purified from tail tips of WT and Smc2Neo/+ mice and digested with MfeI. Southern blot analysis was performed using the Smc2 probe. Successful targeting would give rise to a 10.8-kb fragment, which was shorter than the size of WT (19.1 kb). Expected band pattern was indeed observed from Smc2Neo/+ genomic DNA, indicating correct targeting of the locus. (C) Genomic DNA was subjected to PCR analysis using specific primers as shown in (A). Expected sizes of PCR products were detected for all genotypes, thereby confirming correct targeting. (D) Frozen sections of embryonic brains were immunolabeled with an antibody against SMC2, and stained with Hoechst. The fluorescent intensity of SMC2 was greatly reduced in the VZ at E13.5. The data shown are from a single representative experiment out of three repeats. Sections from three different embryos of each genotype were analyzed. Bar, 50 µm. (PDF) [file pgen.1004847.s003.pdf]

A

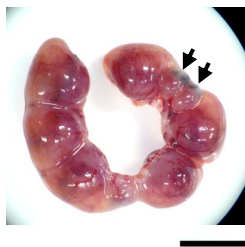

B

| E12.5         | +/+ | +/ $\Delta$ | $\Delta/\Delta$ | total | P value |
|---------------|-----|-------------|-----------------|-------|---------|
| <i>Smc2</i>   | 14  | 25          | 0               | 39    | 0.0014  |
| <i>Ncaph</i>  | 7   | 18          | 0               | 25    | 0.0125  |
| <i>Ncaph2</i> | 13  | 19          | 0               | 32    | 0.0029  |

C

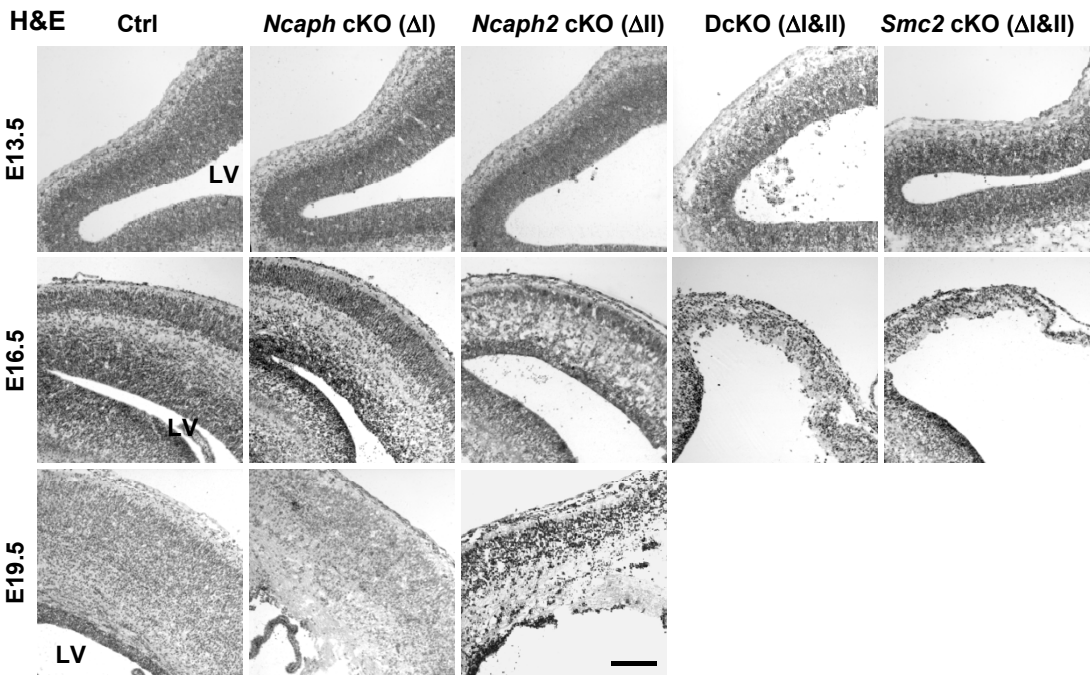

D

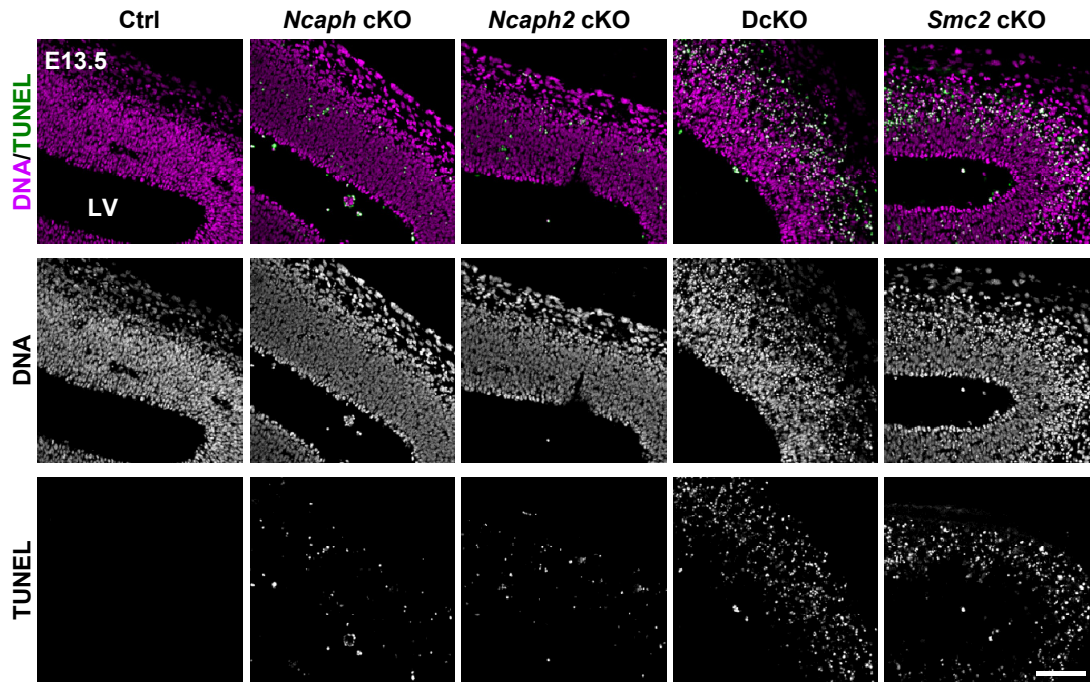

Supplement: S4 Figure — Condensins I and II are both essential for early embryonic development and cortical development. (A) Conventional knockout mice were generated by crossing conditional knockout mice with transgenic mice expressing Cre recombinase ubiquitously. Heterozygotes bearing deletions were mated with each other, and the uterus of female mice was checked for the presence of homozygotes at E12.5. In a representative uterus shown here, empty deciduae (indicated by the arrows) were observed that would have contained homozygotes. Bar, 10 mm. (B) Genotypes of living embryos were determined by PCR analysis. None of the living embryos were judged to be homozygotes (Δ/Δ), suggesting that the homozygotes had disappeared after their implantations. P value was obtained from Chi-squared test, indicating significant deviation from an expected Mendelian ratio. (C) Frozen sections of embryonic brains at the stages indicated were stained with hematoxylin and eosin (H&E). Subtle if any defects were apparent at E13.5 in all mutant mice. By E16.5, however, the brain structures became highly disorganized in DcKO and Smc2 cKO mice. Although morphological defects were relatively mild in Ncaph cKO and Ncaph2 cKO mice at E16.5, the number of cells in the cortex seemed decreased. By E19.5, disorganized cerebral cortices became apparent in both Ncaph cKO and Ncaph2 cKO mice. The data shown are from a single representative experiment out of two repeats. Sections from two different embryos of each genotype were analyzed. Bar, 200 µm. (D) Frozen sections of embryonic brains at E13.5 were subjected to a TUNEL assay to detect apoptotic cell death. DNA was counterstained with Hoechst. Whereas Ncaph cKO and Ncaph2 cKO mice displayed a mild increase in apoptotic cells, massive apoptosis was detected in DcKO and Smc2 cKO mice. The data shown are from a single representative experiment out of two repeats. Sections from two different embryos of each genotype were analyzed. Bar, 100 µm. (PDF) [file pgen.1004847.s004.pdf]

**A**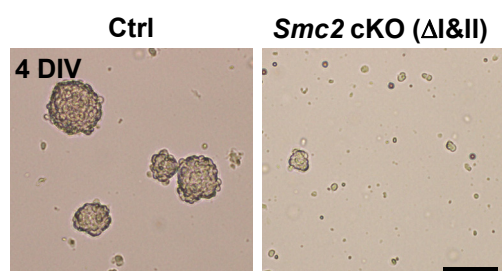**C**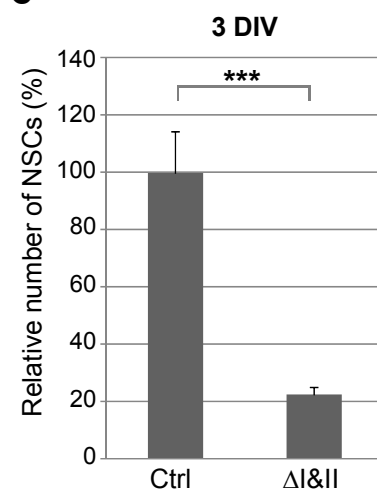**B**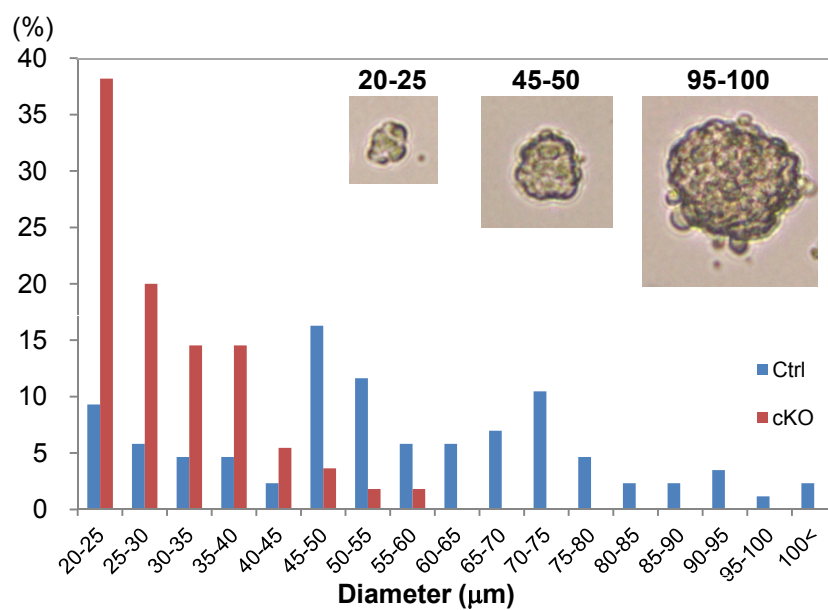**D**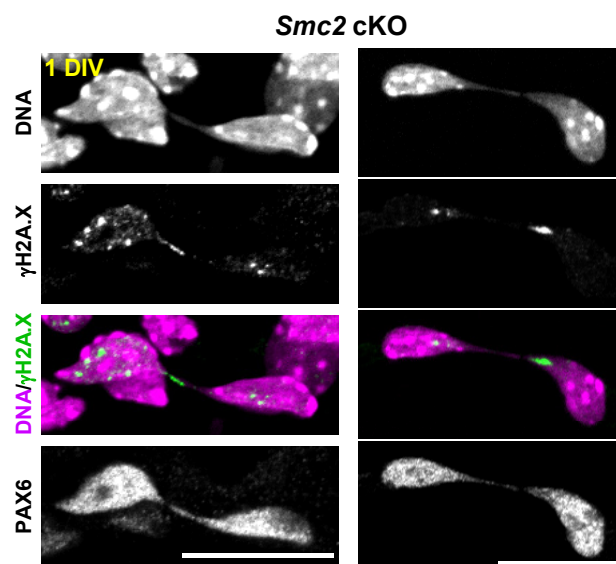

Supplement: S5 Figure — Condensins I and II ensure NSC proliferation in culture. (A) Cells were obtained from the cerebral cortex at E13.5 in control and Smc2 cKO mice, and cultured in vitro for 4 d (4 DIV). The data shown are from a single representative experiment out of three repeats. Bar, 100 µm. (B) Neurospheres formed after 4-d culture were classified based on their diameter, and plotted. The insets indicate representative neurospheres with different diameters (µm). Neurospheres from Smc2 cKO mouse (red) were smaller than those from control mouse (blue). (C) The same number of cells from each brain was plated in culture dishes. After allowing neurosphere to form for 3 d (3 DIV), NSCs are dissociated and their numbers were scored. Data were obtained from three independent cultures and normalized to the mean number of NSCs from control mice as 100%. Bars indicate the mean and SD. *** P<0.001 (t-test). The number of NSCs was dramatically decreased in Smc2 cKO, suggesting their defects in cell proliferation. (D) Cells obtained from the cerebral cortex at E13.5 were cultured for 1 d on coverslips (1 DIV) and immunolabeled with antibodies against PAX6 and γH2A.X. DNA was counterstained with Hoechst. γH2A.X-positive chromatin bridges were observed in PAX6-positive NSCs derived from Smc2 cKO brains, which was consistent with the data obtained in vivo. The data shown are from a single representative experiment out of two repeats. Bar, 20 µm. (PDF) [file pgen.1004847.s005.pdf]

**A**

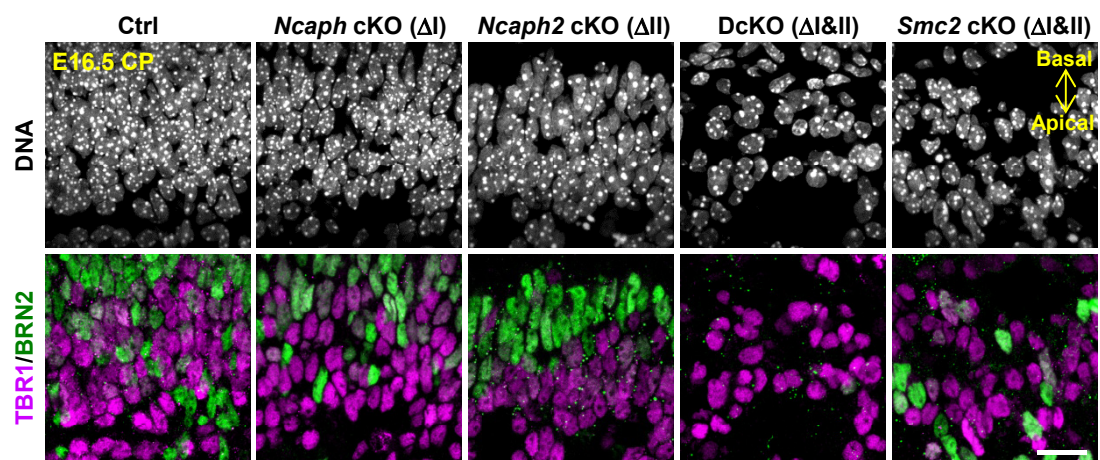

**B**

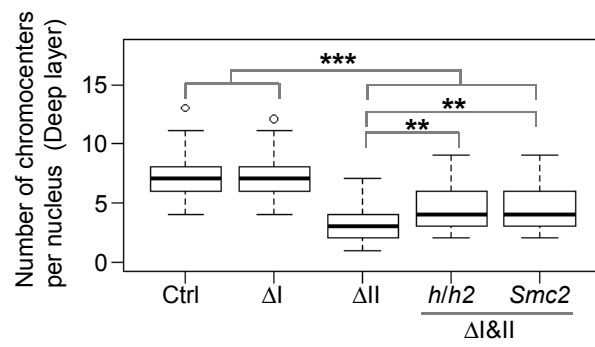

Supplement: S6 Figure — Condensin II prevents hyperclustering of chromocenters in neurons, too. (A) Frozen sections of embryonic brains at E16.5 were fluorescently labeled with antibodies against subtype-specific markers (TBR1 [for deep-layer neurons] and BRN2 [for upper-layer neurons]). DNA was counterstained with Hoechst. Shown here are images focused on TBR1-positive, deep-layer neurons. Hyperclustered chromocenters in neurons were detected in Ncaph2 cKO, DcKO and Smc2 cKO mice, but not in control or Ncaph cKO mice. The data shown are from a single representative experiment out of two repeats. Sections from two different embryos of each genotype were analyzed. Maximum intensity projections are shown. Bar, 20 µm. (B) The numbers of chromocenters per nucleus were measured in TBR1-positive neurons in the cortical plate and plotted. Data were obtained from 50 nuclei. ** P<0.01, *** P<0.001 (t-test with a Holm correction for multiple comparisons). The numbers of chromocenters per nucleus were significantly decreased in Ncaph2 cKO, DcKO and Smc2 cKO mice compared to control and Ncaph cKO mice, implicating condensin II-specific functions for properly organizing nuclear architecture. (PDF) [file pgen.1004847.s006.pdf]

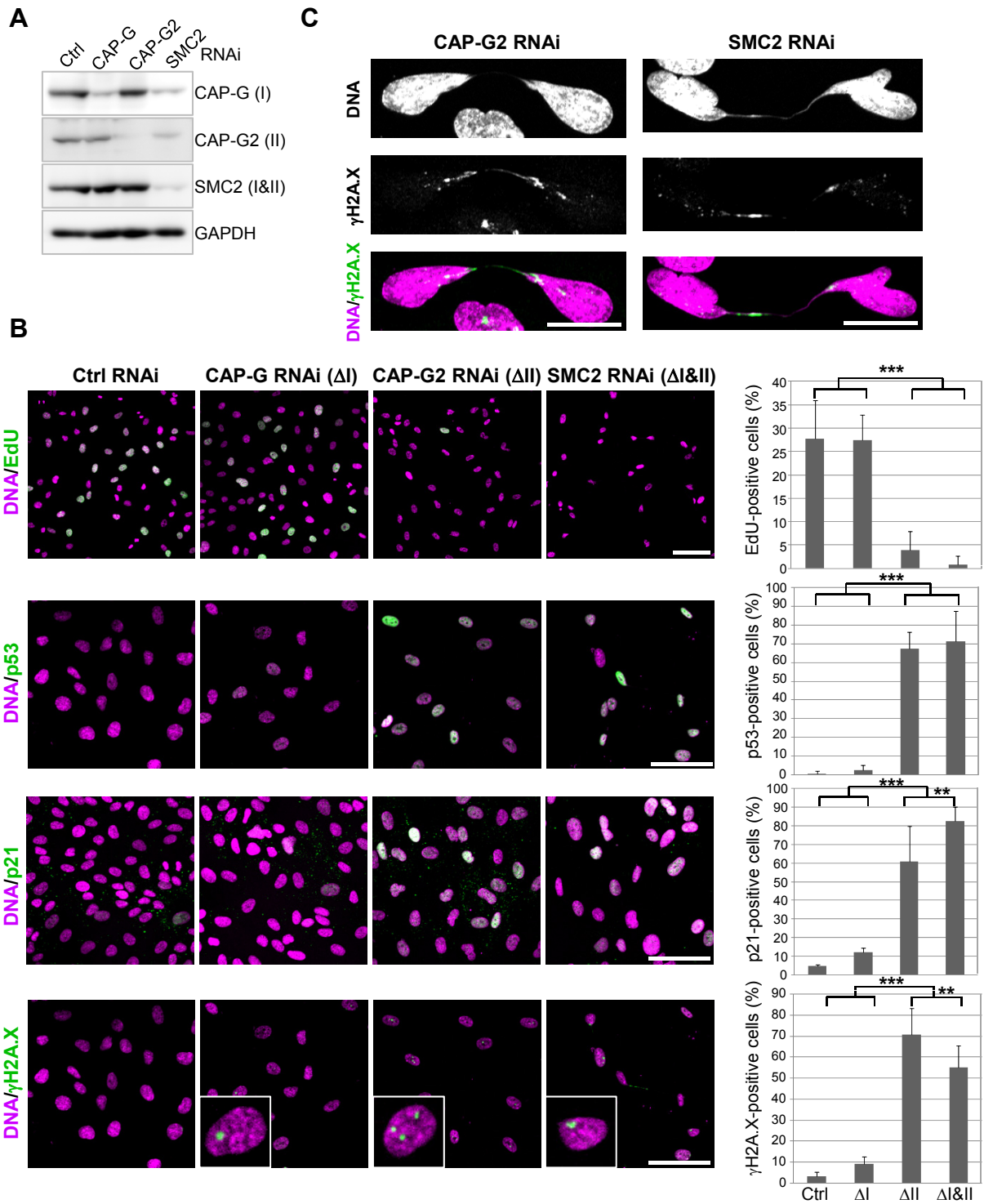

Supplement: S7 Figure — Cell type-dependent contributions of condensins I and II to cell proliferation. (A) RPE-1 cells were treated twice with the siRNAs indicated, plated on culture dishes, and cultured for 1 d. The cells were then harvested and analyzed by western blotting using antibodies against human condensin subunits (CAP-G, CAP-G2 and SMC2) and GAPDH (an internal control). (B) The same set of cells treated with the siRNAs was plated on coverslips and cultured for 2 d. The cells were pulse labeled with 5-ethynyl-2′-deoxyuridine (EdU) before fixation (first row). The percentages of EdU-positive cells were measured, and plotted in the right. RPE-1 cells depleted of CAP-G or SMC2 were barely positive for EdU labeling, whereas CAP-G-depleted cells did not show a significant reduction of EdU-positive cells. Alternatively, the same set of the siRNA-treated cells was immunolabeled with antibodies against p53 (second row), p21 (third row) and γH2A.X (forth row). The percentages of cells with nuclear p53 or with p21 or with γH2A.X foci were measured and plotted in the right. Cells depleted of CAP-G2 or SMC2 displayed high levels of p53-positive, p21-positive and γH2A.X-positive cells, suggesting strongly that loss of condensin II causes DNA damage, p53 nuclear accumulation and p21 up-regulation. These results suggest that depletion of condensin II causes p21-induced cell cycle arrest in RPE-1 cells. All data were obtained from six independent fields from two different cultures. The bars indicate the mean and SD. *** P<0.001 (t-test with a Holm correction for multiple comparisons). The panels shown are from a single representative experiment out of three repeats. Bars, 100 µm. (C) γH2A.X-positive chromatin bridges were observed in cells depleted of CAP-G2 or SMC2, but not of CAP-G (not shown). The panels shown are from a single representative experiment out of three repeats. Bars, 10 µm. (D) RPE-1 cells were treated twice with the siRNAs indicated, plated on culture dishes, and cultured for [file pgen.1004847.s007.pdf]
