# Supplementary material for: Overlapping and Non-overlapping Functions of Condensins I and II in Neural Stem Cell Divisions
Source: PLoS Genet. 2014 Dec 4;10(12):e1004847. doi: 10.1371/journal.pgen.1004847 (PMC4256295; doi:10.1371/journal.pgen.1004847)
Supplement: S1 Table — Primer sequences used in this study. (PDF) [file pgen.1004847.s008.pdf]

For genotyping PCRs

gene	primer	sequence (5' to 3')
Ncaph	Ncaph_f1	AGCCCCCGTGAGAGACTCAC
	Ncaph_f3	CGCTATCTCGCTGCTAAGAGATG
	Ncaph_r1	GTGAAGATGCACAGGACAGCAC
Ncaph2	Ncaph2_f1	GCCTCTGTCTTTGAGAGCTGGAG
	Ncaph2_r1	GTGGCGGCATGTGCCTGTAAG
	Ncaph2_r4	CAGGCAGCAGATGGAGCTCC
Smc2	Smc2_f1	CAGGGGCAATGAACGAGGTCAG
	Smc2_f2	GAAGCTCTCTTGGCCAAGTTTCCC
	Smc2_r2	GGCGGATTTCTGAGTTCGAGGCC

For probe synthesis

probe	primer	sequence (5' to 3')
Ncaph probe	Ncaph_PB_fwd1	CCTGCACAATGTTCTGGTATGTCC
	Ncaph_PB_rvs1	GTTTTGAGTCTACGCGCGAGTG
Ncaph2 probe	Ncaph2_PB_fwd1	GCAGCTATGTGCCGACGTATACTC
	Ncaph2_PB_rvs1	CCGCTATGTGCAAGAATTCCACC
Smc2 probe	Smc2_PB_fwd1	GCATGCTCCAGAGGACACGTG
	Smc2_PB_rvs1	CTTCATACTGGCATGGCTACTCTAACAG

For RT-PCRs

target	primer	sequence (5' to 3')
Gapdh	mGapdh_qF1	GGCTGCCCAGAACATCATCCC
	mGapdh_qR1	AGGCCATGCCAGTGAGCTTC
Bax	mBax_qF1	TGGGCTGGACACTGGACTTC
	mBax_qR1	ATGTGGGGGTCCCGAAGTAG
Noxa	mNoxa_qF1	CGCAAAAGAGCAGGATGAGG
	mNoxa_qR1	CAATCCTCCGGAGTTGAGCA
