# Supplementary material for: Overlapping and Non-overlapping Functions of Condensins I and II in Neural Stem Cell Divisions
Source: PLoS Genet. 2014 Dec 4;10(12):e1004847. doi: 10.1371/journal.pgen.1004847 (PMC4256295; doi:10.1371/journal.pgen.1004847)
Supplement: S2 Table — Antibodies used in this study. (PDF) [file pgen.1004847.s009.pdf]

Primary antibodies generated in our lab.

species	antigen	reference #	catalogue #	dilution IHC	ICC	WB	IP
Rabbit	mouse SMC2	17	AfR307-3L	1:500	-	1:1000	1μg
	mouse CAP-G	17	AfR310-4L	-	-	1:1000	1μg
	mouse CAP-H2	17	AfR427-3L	1:1000	-	1:1000	1μg
	human SMC2	13	AfR50-4	-	-	1:1000	-
	human CAP-G	13	AfR55-5L	-	-	1:1500	-
	human CAP-G2	13	AfR210-3L	-	-	1:1000	-
rat	mouse CAP-H	17	AfRt10-3	1:100	-	-	-

Commercially available primary antibodies

species	antigen	manufacturer	catalogue/clone #	dilution IHC	ICC	WB
mouse	SOX2	R&D systems	MAB2018	1:100	-	-
	GAPDH	Millipore	MAB374	-	-	1:1000
	γH2A.X	BioLegend	613401/2F3	-	1:250	-
	H3S10ph	CST	9706/6G3	1:500	-	-
Rabbit	53BP1	abcam	ab21083	1:200	-	-
	Cleaved Caspase 3	CST	9664/5A1E	1:100	-	-
	H3K9me3	Millipore	07-523	1:500	-	-
	Nucleolin	abcam	ab22758	1:250	-	-
	p21	Santa Cruz	sc-397	-	1:100	-
	p53	Santa Cruz	sc-62423	1:100	1:100	-
	PAX6	Millipore	AB2237	1:200	1:200	-
	TBR1	abcam	ab31940	1:200	-	-
goat	Lamin B	Santa Cruz	sc-6217	1:500	-	-
	BRN2	Santa Cruz	sc-6029	1:100	-	-
rat	BrdU	abcam	ab6326/rat BU1_75	1:500	-	-

Commercially available secondary antibodies

antigen	conjugation	manufacturer	catalogue/clone #	dilution IHC	ICC	WB
rabbit IgG	Alexa Fluor 488	Life Technologies	A21206	1:500	1:500	-
	Alexa Fluor 555	Life Technologies	A21206	1:500	1:500	-
	HRP	Vector	PI-1000	-	-	1:5000
mouse IgG	Alexa Fluor 488	Life Technologies	A11001	1:500	1:500	-
	Alexa Fluor 568	Life Technologies	A11031	1:500	1:500	-
	HRP	Vector	PI-2000	-	-	1:5000
goat IgG	Alexa Fluor 488	Life Technologies	A11055	1:500	1:500	-
rat IgG	Alexa Fluor 488	Life Technologies	A11006	1:500	1:500	-

IHC, immunohistochemistry; ICC, immunocytochemistry; WB, western blot; IP,immunoprecipitation
